# COVID-19 Lung Pathogenesis in SARS-CoV-2 Autopsy Cases 

**DOI:** 10.3389/fimmu.2021.735922

**Published:** 2021-10-04

**Authors:** Silvana Valdebenito, Simon Bessis, Djillali Annane, Geoffroy Lorin de la Grandmaison, Elisabeth Cramer–Bordé, Brendan Prideaux, Eliseo A. Eugenin, Morgane Bomsel

**Affiliations:** ^1^ Department of Neuroscience, Cell Biology and Anatomy, University of Texas Medical Branch (UTMB), Galveston, TX, United States; ^2^ Service des Maladies Infectieuses, Centre Hospitalier Universitaire Raymond Poincaré, AP-HP, Garches, France; ^3^ Intensive Care Unit, Raymond Poincaré Hospital (AP-HP), Paris, France; ^4^ Simone Veil School of Medicine, Université of Versailles, Versailles, France; ^5^ University Paris Saclay, Garches, France; ^6^ Department of Forensic Medicine and Pathology, Versailles Saint-Quentin Université, AP-HP, Raymond Poincaré Hospital, Garches, France; ^7^ University of Versailles Saint Quentin en Yveline, Versailles, France; ^8^ Laboratory of Mucosal Entry of HIV-1 and Mucosal Immunity, Department of Infection, Immunity, and Inflammation, Institute Cochin, CNRS UMR 8104, INSERM U1016, University of Paris, Paris, France

**Keywords:** COVID-19, SARS – CoV – 2, immune, immune activation, lung

## Abstract

Severe acute respiratory syndrome coronavirus 2 (SARS-CoV-2) is a major public health issue. COVID-19 is considered an airway/multi-systemic disease, and demise has been associated with an uncontrolled immune response and a cytokine storm in response to the virus. However, the lung pathology, immune response, and tissue damage associated with COVID-19 demise are poorly described and understood due to safety concerns. Using post-mortem lung tissues from uninfected and COVID-19 deadly cases as well as an unbiased combined analysis of histology, multi-viral and host markers staining, correlative microscopy, confocal, and image analysis, we identified three distinct phenotypes of COVID-19-induced lung damage. First, a COVID-19-induced hemorrhage characterized by minimal immune infiltration and large thrombus; Second, a COVID-19-induced immune infiltration with excessive immune cell infiltration but no hemorrhagic events. The third phenotype correspond to the combination of the two previous ones. We observed the loss of alveolar wall integrity, detachment of lung tissue pieces, fibroblast proliferation, and extensive fibrosis in all three phenotypes. Although lung tissues studied were from lethal COVID-19, a strong immune response was observed in all cases analyzed with significant B cell and poor T cell infiltrations, suggesting an exhausted or compromised immune cellular response in these patients. Overall, our data show that SARS-CoV-2-induced lung damage is highly heterogeneous. These individual differences need to be considered to understand the acute and long-term COVID-19 consequences.

## Introduction

Coronavirus disease 2019 (COVID-19) is a rapid and emerging pandemic disease caused by the severe acute respiratory syndrome coronavirus-2 (SARS-CoV-2). Current medical management is largely supportive, with no well-tested therapy available despite several efforts to use (hydroxy)chloroquine, dexamethasone, and remdesivir as well as neutralizing monoclonal antibodies treatments (1–5). Further, several vaccines efficiently prevent the most severe COVID-19 symptoms, but the pathogenesis of the virus is still not fully understood.

Overall, it is accepted that SARS-CoV-2 primarily affects the respiratory system, although other organs are also involved (6–10). The most common symptoms are fever, dry cough, dyspnea, headache, dizziness, vomiting, diarrhea, and generalized weakness (11–13). However, clinically it is widely recognized that COVID-19 symptoms are extremely heterogeneous (12). Epidemiological studies have shown that mortality rates are higher in the elderly and people with existing comorbidities such as high blood pressure, diabetes, obesity, and impaired respiratory conditions (7, 11, 14, 15). Moreover, the variability of the disease and the effects of existing comorbidities are not fully understood. It remains urgent to comprehend COVID-19 pathogenesis to design efficient treatments for preventing or reducing acute and long-lasting damage.

Detailed studies using autopsy tissues have shown that COVID-19 pathogenesis was associated with thrombosis (micro and macro-vasculature), compromised blood vessel integrity, inflammation, fibrin structures, occlusion of alveolar spaces, multinucleation, and interferon related responses accompanied with the viral presence (Spike (S) protein and viral RNA) (16–20). However, the viral replication time course in lung tissues and other organs remains controversial (16–23). As most COVID-19 reports were limited to the evaluation of peripherical blood markers, *in vitro* models (19, 24–30), and gross lung anatomical analysis and basic histology assessments, large 3-dimensional (3D), multi-host and viral analyses are still lacking.

In the present study, using a multiparametric immuno-morphological analysis, we now report an overwhelming lung damage heterogeneity among different individuals and even within the same individual. However, the lung damage were consistent with deadly conditions revealing the fast and destructive nature of SARS-CoV-2. The identification of these heterogeneous mechanisms should provide a new understanding of COVID-19 pathogenesis.

## Materials and Methods

### Reagents

All reagents were purchased from Sigma (St. Louis, MO) unless indicated otherwise. Dyes and secondary antibodies were obtained from Thermo-Fisher (Waltham, MA). RNAscope 2.5 HD Detection of s-sense COVID-19 for RNA detection was used (Hayward, CA). Antibodies for macrophages (Iba-1, Ab5076), lymphocytes (CD3, ab11089), endothelial cells (Von Willebrand factor, ab194405), epithelial cells (EpCam, ab7504), myeloperoxidase (MPO, ab25989), CD8 (CD8, ab22378), CD20 (B cells, ab9475), and smooth muscle actin (SMA, ab21027) were obtained from Abcam, MA. Vimentin (sc-52721) from Santa Cruz (Santa Cruz, CA) and the antibody for SARS protein M (APO90991su-n) were obtained from Origene (Rockville, MD). All experiments were performed under the University of Texas Medical Branch (UTMB) and the NIH regulations.

### Study Participants and Ethical Issues

Large lung samples were sampled from 11 rapid autopsies with confirmed SARS-CoV-2 infection at the Forensic Medicine and Pathology, Versailles Saint-Quentin University, AP-HP, Raymond Poincaré Hospital, Garches, France, and UTMB. This non-interventional study was approved by the institutional review board of the ethical committee for research (CER) of the University of Paris Saclay (CER-Paris-Saclay-2020- 050) and conformed to the principles outlined in the Declaration of Helsinki. All patients were confirmed positive for SARS-CoV-2 RNA by RT-PCR at the hospital using blood samples. Non-COVID-19 control lung biopsies were obtained from the pathological anatomy service at UTMB. No personal information from the corresponding donors was collected. Lung samples corresponded to discarded tissues from pathological analysis of COVID-19 infected (n=11), border healthy-carcinoma lung tumors (n=4), and resected lung areas with Tuberculosis (n=2). Sample collection, processing, and laboratory testing complied with World Health Organization guidance. Patient clinical data are summarized in **Table 1**.

**Table 1:** Patient Sample Information.

Patient	Sex	Age	Sample Origin	ICU days	Days until demise	BMI index	Pre-existing condition	Treatment	Cause of death
1-COVID-19	F	82	France	11	17	24.1	N.A.	N.A.	Multivisceral failure
2-COVID-19	M	51	France	10	20	34.5	Small airways obstruction/Leukemia	AINS	Pulmonary embolism
3-COVID-19	M	80	France	4	11	23.9	Meningioma	N.A.	Multivisceral failure
4-COVID-19	F	59	France	13	18	28.4	Asthma	Corticoids	Pulmonary embolism
5-COVID-19	M	51	France	11	18	38	Small airways obstruction/Arthritis	N.A.	Massive Pulmonary embolism
6-COVID-19	F	76	France	11	14	31.2	N.A.	Methotrexate	Septic shock
7-COVID-19	M	51	France	53	30	35.1	N.A.	N.A.	Pulmonary embolism
8-COVID-19	N.A.	N.A.	France	N.A.	N.A.	N.A.	N.A.	Home	N.A.
9-COVID-19	N.A.	N.A.	France	N.A.	N.A.	N.A.	N.A.	Home	N.A.
10-COVID-19	N.A.	N.A.	France	N.A.	N.A.	N.A.	N.A.	Home	N.A.
11-COVID-19	N.A.	N.A.	France	N.A.	N.A.	N.A.	N.A.	Home	N.A.
1-LC	N.R.	N.A.	USA	N.A.	N.A.	N.A.	Lung cancer	N.A.	N.A.
2-LC	N.R.	N.R.	USA	N.A.	N.A.	N.R.	Lung cancer	N.R.	N.R.
3-LC	N.R.	N.R.	USA	N.A.	N.A.	N.R.	Lung cancer	N.R.	N.R.
4-LC	N.R.	N.R.	USA	N.A.	N.A.	N.R.	Lung cancer	N.R.	N.R.
1-TB	M	58	USA	N.A.	N.A.	N.R.	TB	N.A.	N.A.
2-TB	M	65	USA	N.R.	N.A.	N.R.	TB	N.A.	N.A.

COVID-19, Coronavirus disease 19; LC, Lung Carcinoma; TB, tuberculosis; N/A, not applicable; N/R, not register; F, female; M, Male; AINS, non-steroidal anti-inflammatory drugs.

### qRT–PCR to Detect SARS-CoV-2

The throat swab, sputum from the upper respiratory tract, and blood were collected from patients after hospitalization or in-home death for SARS-CoV-2 RNA detection.

### Histology

The resected lung (COVID-19 and lung carcinoma) was immediately transferred to a biological class 2 cabinet and dissected into large pieces. Samples were fixed in 10% neutral buffered formalin for at least 24 h, dehydrated, and paraffin-embedded for further analysis. A minimum of 15-20 sequential sections (20-50 µm each) were cut from each tissue to perform histology, confocal microscopy, 3D-reconstruction of large pieces of tissue, deconvolution, and image analysis, as we described (31–34). Sections from each block were prepared for hematoxylin and eosin (H&E) and trichrome staining, and the stained slides were imaged at 20× using a Hamamatsu slice scanner NanoZoomer 2.0RS. This equipment allows us to scan large tissue areas in the X, Y, and Z planes. An experienced pathologist reviewed the images and 3D-reconstructions.

### Immunofluorescence and Confocal Microscopy

As we described recently, large lung tissue sections were cut and processed (up to 5 cm) (35). Briefly, in addition to deparaffination, we eliminated or reduced autofluorescence using a light source in the green and red channels (35). Tissues were then incubated in Sudan Black and sodium borohydride to reduce further autofluorescence (35). Lung tissue sections were treated with the RNAscope Multiplex Fluorescent Reagent Kit v2 Assay protocol (ACDbio), following manufacturer instructions. The procedure includes multiple steps of sample pretreatment, including RNAscope target retrieval reagent for 15 min, RNAscope protease plus for 15 min, hybridization (RNA probe V-nCOV2019-S-sense, specific for SARS-CoV-2), and signal development (TSA Plus cyanine 5 fluorophore, Opal 690). Each step was followed by two successive washes with 1X wash buffer. Samples were then incubated in blocking solution for 2 hours at room temperature, followed by overnight incubation at 4°C with diluted primary antibodies. Cells were then washed several times with PBS and incubated with the appropriate secondary antibody for at least 2 hours at room temperature, followed by an additional wash in PBS. Tissues were examined using an A1 Nikon confocal microscope with spectral detection (Nikon, Tokyo, Japan). Antibody specificity was confirmed by replacing the primary antibody with a non-specific myeloma protein of the same isotype or non-immune serum, as we described (35–37). Analyses of the 3D-reconstruction and deconvolution were performed using NIS Elements (Nikon, Japan).

### Image Analysis of Correlative Histology and Confocal Microscopy

Three-D-reconstruction and deconvolution from 15 to 20 successive optical sections (20-50 µm) were performed, resulting in an extensive area analysis in the X, Y, and Z-axis. To analyze and quantify the abundance of the signal, the number of positive pixels and their intensity in different cell populations were measured in specific regions of interest. Controls with nonspecific IgGs and non-immune serums, as well as irrelevant probes, were included. The autofluorescence of the tissues also was decreased, as described above.

### Statistical Analysis

Data were analyzed using Origin 8.1 (Northampton, MA, US). For single comparisons, Student’s t-test was used. For multiple comparisons, ANOVA was used; *p* values of < 0.05 were considered significant.

## Results

### Analysis of Large Lung Areas Obtained From Fatal COVID-19 Cases

The COVID-19 cohort had an average stay of 28 ± 21 days in the Hospital before demise (see **Table 1**). Comorbidities such as diabetes were not present, and the obesity range was mild (see **Table 1**). All individuals analyzed had mild hypertension, and no lung-associated comorbidities were present. To ensure an unbiased assessment, all samples were received and analyzed blindly. After all the data was acquired, the clinical and COVID-19 status was requested to assure proper scientific rigor. Gross histological analysis by Hematoxylin and Eosin and trichrome staining showed significant pathology heterogeneities among individuals. Overall (see details below), the combined histology and confocal imaging identified at least three different types of pathology associated with deadly COVID-19 infection. We will first describe the histology findings prior to providing a more complex evaluation from 3D-reconstructions, deconvolution, and image analysis of the same areas obtained using correlative microscopy.

Additionally, we observed another fourth type of pathology not described in the manuscript due to the total loss of alveolar structures with caseous necrosis. This later condition was not considered or quantified due to the extensive lung tissue destruction, and therefore data are not represented and not included in the present report. COVID-19 samples were compared to normal, carcinoma, and long-term tuberculosis lung tissue.

### COVID-19-Induced Phenotype 1: Enhanced Coagulation/Hemorrhage

**Figure 1:**
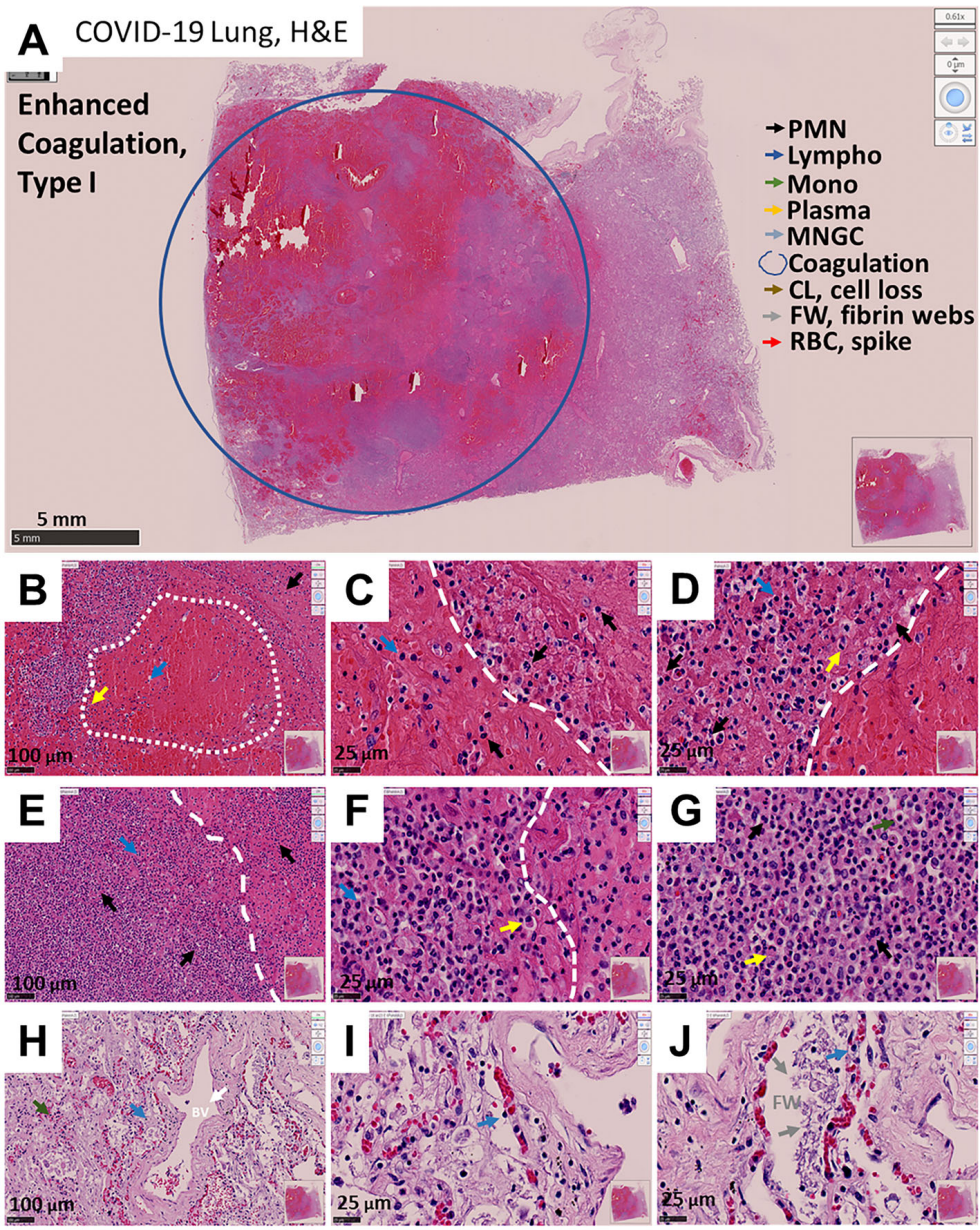
Representative microphotograph of eight serial sections of lung obtained from a deadly case of COVID-19 with hemorrhagic events and immune infiltration. **(A)** Representative H&E-stained images of lung classified as enhanced coagulation or type I damage. Low magnification allows us to appreciate the degree of damage and the large hemorrhagic events. The blue circle indicates the area with significant hemorrhagic and hemolysis events. Bar: 5 mm. **(B–D)** Correspond to higher magnification to denote the interphase between blood and the immune rim around the lesions (see dotted line). Bar 100 µm and 25 µm. **(E–G)** Correspond to the immune infiltration around the lesions. Bar 100 µm and 25 µm. **(H–J)** Represent areas outside of the hemorrhagic areas but with intravascular retention of red blood cells. Observe the fibrosis and formation of intravascular fibrin webs. Due to these characteristics, we named this phenotype enhanced coagulation. As indicated in **(A, B–J)** arrows of different colors indicated different cell types, coagulation areas, cell loss (CL), Fibrin webs (FW), and red blood cells with spiked membrane. n = 4 different individuals with 15-20 serial sections.

In this phenotype, large numbers of blood products such as leukocytes and red blood cells were accumulated within the alveolar space and blood vessels (**Figure 1A**, EC, low magnification, blue circle denotes a large lesion). Further, hemorrhagic events were associated with the destruction of alveolar walls and blood vessels with minimal immune cell infiltration within the lesions (See **Figures 1B–D**, the arrow indicates different cell types; see arrow colors in A). Most immune cells accumulate around the hemorrhagic lesions (**Figures 1E–G**, the dotted line represents the separation between the lesion and the immune cell rim), suggesting an immune “containment” or “barrier” to prevent further lung compromise. This phenotype is similar to the tissue damage reported for *Mycobacterium tuberculosis-*induced immune response around granulomas (38, 39). However, in COVID-19 cases, the size of the lesions was bigger (in the range of cm) and poorly organized. The main immune cell types observed in the hemorrhagic lesions corresponded to polymorphonuclear (PMN, back arrows), lymphocytes (Lympho, blue arrows), monocytes (Mono, green arrows), plasma cells (yellow arrows), and normal shaped and spiky red blood cells (RBC, spike, or acanthocytes, red arrows). The presence of these spiking RBC has been described in several severe non-infectious diseases, including liver, neural, lipid dysregulation, spleen loss, and kidney damage (40–47), suggesting a systemic effect of COVID-19. In these COVID-19 lung tissues, the separation between the hemorrhagic and immune rim was clear and remarkable (**Figures 1C, E, F**).

In lung areas with minimal hemorrhagic events, intravascular coagulation, loss of the pneumocyte monolayer (type I and II) integrity, blood vessel continuity, and exacerbated fibrosis, as well as intravascular fibrin web formation, were commonly observed (**Figures 1H–J**, FW see arrows). Lung regions around these hemorrhagic lesions were surrounded by an immune rim and showed signs of fibrosis (**Figures 1H–J**). However, independent of the area analyzed, alveolar spaces were clotted with blood products or exacerbated fibrosis. In contrast, analysis of uncompromised lung tissue obtained from individuals during lung carcinoma screening [similar to lungs from uninfected individuals (48, 49)] showed similar histological features of normal lungs with some tumor cells in the alveolar space (**Supplemental Figure 1**. Note that all magnifications of the image shown are identical to that in **Figure 1**). Furthermore, COVID-19 and control lung tissues showed remarkable differences in the free or empty alveolar space required for efficient gas exchange, intravascular or extravascular coagulation, pneumocyte, and endothelial distribution, and immune infiltration (**Supplemental Figure 1**).

To examine the fibrosis degree in COVID-19 cases, we performed trichrome staining (normally used to evaluate the accumulation of collagen and other extracellular matrix proteins for extended periods). The intense and widely distributed trichrome staining in COVID-19 cases compared to the fibrosis present in lung carcinoma (**Supplemental Figure 2**) exceeded our expectations for a disease developed in days to weeks, suggesting an exacerbated mechanism of ECM secretion and an accumulation in all COVID-19 cases analyzed (**Supplemental Figure 3**).

### COVID-19-Induced Phenotype 2: Immune Infiltration With Minimal Hemorrhagic Events

**Figure 2:**
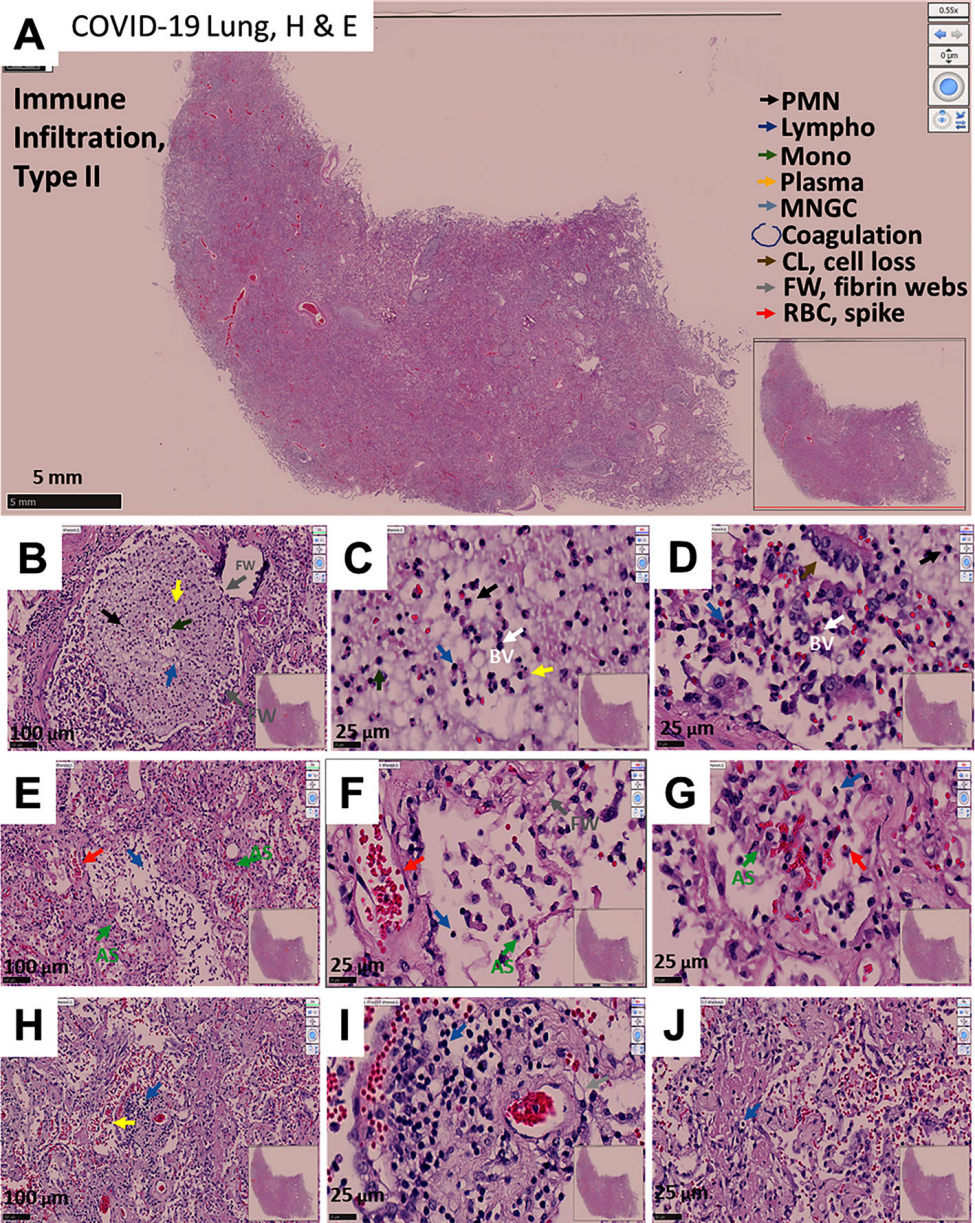
Representative microphotograph of eight serial sections of lung obtained from a deadly case of COVID-19, with immune infiltration without hemorrhagic events. **(A)** Representative H&E-stained lung classified images as immune infiltration. Low magnification allows us to appreciate the degree of damage, fibrosis, and intravascular coagulation webs trapping immune cells within the blood vessels. Bar: 5 mm. **(B–D)** Correspond to higher magnification to denote the intravascular clots and webs. Bar 100 µm and 25 µm. **(E–G)** Correspond to fibrotic alveolar areas. Bar 100 µm and 25 µm. **(H–J)** represent fibrotic alveolar areas with significant immune infiltration. Note the fibrosis and formation of intravascular fibrin webs. As indicated in A, arrows of different colors indicate representative cell types, areas with coagulation, cell loss (CL), Fibrin webs (FW), and red blood cells with spikes (RBCs). n = 4 different individuals with 15-20 serial sections.

The second phenotype of lung pathology observed in deadly cases of COVID-19 corresponded to a strong immune infiltration with mild preservation of alveolar walls but clotted blood vessels. This was accompanied by an increased number of intra-vascular/intra-alveolar fibrin webs compared to phenotype 1, but without major hemorrhagic events as indicated at the low magnification (compare to **Figure 2A** to **Figure 1A**). Higher magnification revealed alveolar wall hyperplasia, but more striking was the loss of pneumocytes (type I and II, **Figures 2B–G**) and extensive fibrosis (later confirmed by trichrome staining). Also, there was the intra-vascular/intra-alveolar formation of fibrin webs trapping leukocytes and lung cell types (**Figures 2B–D**).

A strong and widespread immune response was observed, leaving some free alveolar space for gas exchange, although likely resulting in acidification and poor gas exchange (**Figures 2E–J**). The main immune cell types observed were polymorphonuclear (PMN, back arrows), lymphocytes (Lympho, blue arrows), monocytes (Mono, green arrows), plasma cells (B cells, yellow arrows), and spiked RBCs (red arrows) without the clear organization as described for phenotype 1. However, critical histological features of this phenotype were the alveolar cell loss by multicellular detachment from the basal lamina (CL, cell loss, brown arrow) together with increased numbers and extension of fibrin webs (lead-colored arrows), suggesting a strong immune response in these patients prior to death. A similar loss of multicellular areas of the lung has been described in tuberculosis resulting in lung cavities (50, 51). We also observed the accumulation of spiked RBC, suggestive of an acute systemic dysfunction as described for phenotype 1. In contrast, none of the conditions described above were observed in non-COVID-19 lung tissue obtained from lung carcinoma (LC) (compare to **Supplemental Figure 1**). As shown by Trichrome staining, a significant increase in fibrosis was observed in blood vessels and the alveolar walls in association with web fibrin (**Supplemental Figure 4**). A representative image denoting the loss of multicellular areas of the lung is shown in **Supplemental Figure 4I**. Overall, COVID-19-induced phenotype 2 is characterized by a strong immune disorganized immune response, the lack of hemorrhagic events, intravascular webs (probably due to fibrin), and alveoli loss.

### COVID-19-Induced Phenotype 3: Mixed Conditions

**Figure 3:**
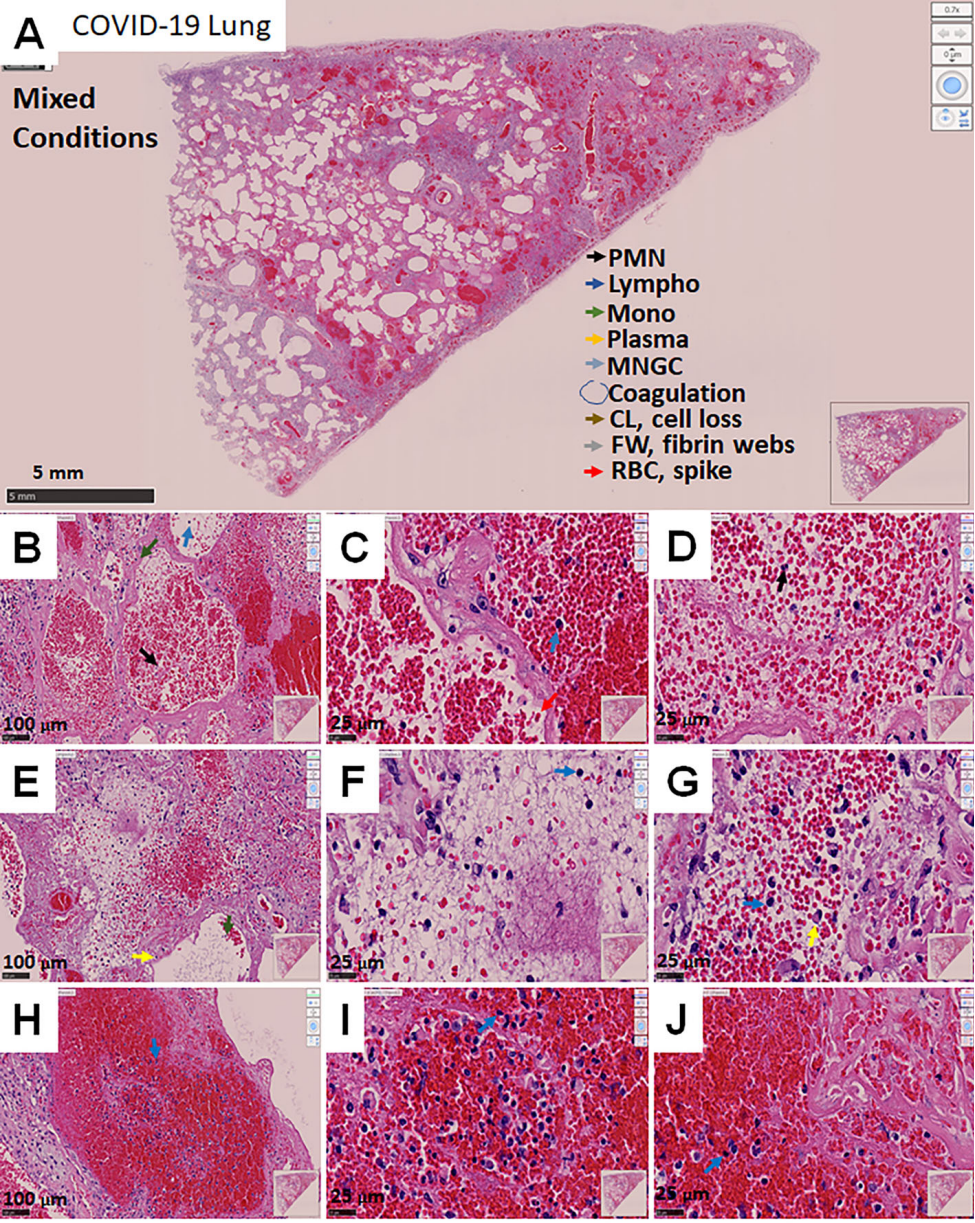
Representative microphotograph of eight serial sections of lung obtained from a deadly case of COVID-19, with mixed conditions, with hemorrhagic lesions, fibrosis, immune infiltration, fibrin webs, and significant lung damage. **(A)** Representative H&E-stained images of lung classified as mixed conditions due to the combination of phenotypes I and II. **(A)** Low magnification allows us to appreciate the degree of damage, hemorrhagic, fibrosis, intravascular coagulation webs trapping immune cells within the blood vessels. Bar: 5 mm. **(B–D)** Correspond to higher magnification to denote the intravascular and hemorrhagic parenchymal lesions. Bar 100 µm and 25 µm. **(E–G)** Correspond to intravascular areas. Bar 100 µm and 25 µm. **(H–J)** represent contained hemorrhagic lesions within the parenchyma. Due to these characteristics, we named this phenotype mixed conditions. As indicated in A, arrows of different colors indicate representative cell types, areas with coagulation, cell loss (CL), Fibrin webs (FW), and red blood cells with spikes (RBCs). n=4 different individuals with 15-20 serial sections.

The third phenotype of lung tissue pathology identified in COVID-19 autopsies corresponded to a mixed phenotype of the two previously described ones with some minor particularities. These cases showed areas with hemorrhagic events (phenotype 1), mild-strong immune infiltration, intravascular web fiber (phenotype 2), and tissue loss more pronounced than phenotypes 1 and 2 (see low magnification picture, **Figure 3A**). In addition, lung cavities with excessive loss of tissue (**Figure 3A**) and strong fibrosis (**Supplemental Figure 5**) had formed as reported for lung cavities in *mycobacterium tuberculosis* long-term infection and damage (50, 51).

Remarkably, this phenotype showed that hemorrhagic events were well contained but lacked a clear immune rim, as observed in the enhanced coagulation phenotype 1 (**Figures 3B–D**). Most RBCs were accumulated inside blood vessels, fibrotic parenchyma, and a fibrin web (**Figures 3E–G**). Some areas showed clear signs of hemolysis and compromised RBC (**Figures 3H–J**, RBC spiked membrane). However, several alveoli were intact in this mixed phenotype (**Figure 3A**). None of the characteristics described for this phenotype were observed in non-COVID-19 lung carcinoma tissues (compared to **Supplemental Figure 1**). Trichrome staining indicated a large accumulation of collagen in vascular areas and parenchymal alveolar areas (**Supplemental Figures 5A–J**), supporting strong fibrosis in the absence of immune infiltration and formation of lung cavities only in this phenotype.

### Quantification of the Immune Cells and Lung Damage in COVID-19 Lungs

**Figure 4:**
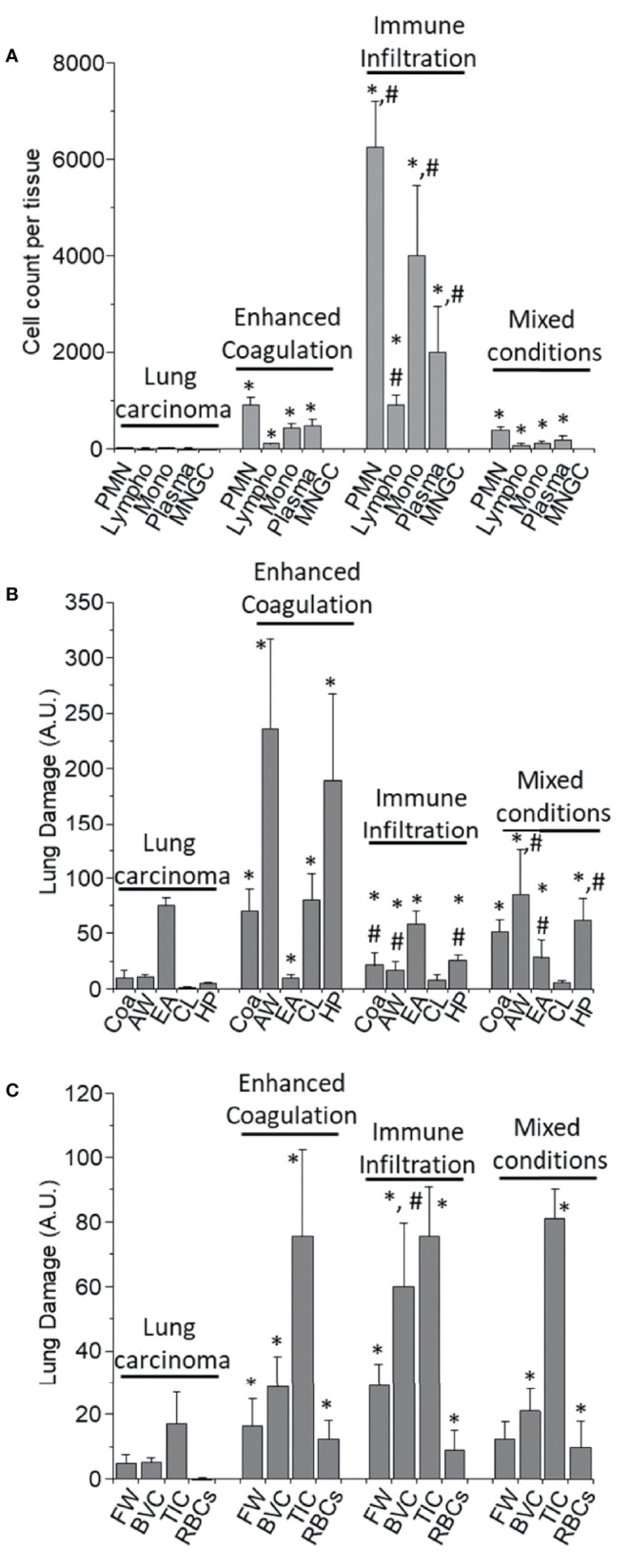
Quantification of the immune infiltration and lung damage. **(A)** H&E tissue slices were used to quantify the numbers and types of immune cells within the different tissues analyzed. We quantified Polymorphonuclears (PMN), Lymphocytes (Lympho), Monocytes (Mono), Plasma cells (Plasma), and multinucleated giant cells (MNGC) in the different COVID-19 pathology phenotypes, enhanced coagulation, immune infiltration, and mixed conditions, as well as in lung carcinoma samples. In lung carcinoma samples, minimal infiltration of PMN, Lympho, Mono, and Plasma was observed. No MNGC was observed in any of the tissues analyzed. In the enhanced coagulation phenotype, PMN infiltration was the prominent cell type infiltrated. Monocytes, Plasma cells, and lymphocytes migration were minimal compared to lung carcinoma (*p ≤ 0.005 compared to lung carcinoma, n = 4 different individuals with 5 sections each). In the Immune infiltration phenotype, a significantly higher lung infiltration was detected compared to lung carcinoma (*p ≤ 0.0001 compared to lung carcinoma, n = 4 different individuals with 5 sections each) and enhanced coagulation (^#^p ≤ 0.007 compared to lung carcinoma, n = 4 different individuals with 5 section each). A similar proportion of cells infiltrate the lung, but also with a minimal lymphocyte infiltration. In the mixed conditions, a similar infiltration than in enhanced coagulation was observed (*p ≤ 0.005 compared to lung carcinoma, n = 3 different individuals with five sections each). No MNGC was observed under any of the conditions examined. **(B)** Quantification of lung damage by examining hemorrhagic/coagulation related lesions (Coa, data expressed in percentage), Alveolar Wall thickness (AW, data expressed as µm), the empty area of the lung (EA, expressed as a percentage), Cell Loss (CL, expressed in percentage and focus mainly in pneumocyte loss into the alveolar space), and hyperplastic Pneumocyte (HP, data expressed as µm). In lung carcinoma, all the aspects examined were similar to the data from multiple publications in healthy conditions. In the enhanced coagulation phenotype (type I), coagulation, alveolar wall, cell loss, and hyperplastic pneumocyte were significantly higher than all the conditions examined. However, a dramatic reduction in the lung’s empty area, a condition required for efficient gas exchange, was detected (*p ≤ 0.00126 compared to lung carcinoma, n = 3 different individuals with five sections each). In the immune infiltration phenotype, significant damage was observed (*p ≤ 0.005 compared to lung carcinoma and ^#^p ≤ 0.007 compared to enhanced coagulation, n = 4 different individuals with 5 sections each). A similar profile than immune infiltration was observed in the mixed conditions with a significant compromise in the EA (^#^p ≤ 0.00125 compared to enhanced coagulation, n = 4 different individuals with five sections each). **(C)** Quantification of the lung damage by determining the area with fibrin webs (FW), Blood vessel collagen (BVC), tissue-associated collagen (TIC), and spiky red blood cells (RBCs) using the H&E and trichrome staining was performed. In lung carcinoma, most data were similar to healthy conditions. In all COVID-19 phenotypes, lung damage was elevated equally, except BVC in the immune infiltration higher than in the enhanced coagulation phenotype (*p ≤ 0.005 compared to lung carcinoma and ^#^p ≤ 0.0002 compared to enhanced coagulation phenotype, n = 13 different individuals with 5 section each).

To quantify the degree of immune infiltration, we counted the total numbers of polymorphonuclear (PMN), lymphocytes (Lympho), monocytes (Mono), plasma cells (Plasma), and multinucleated giant cells (MNGC) in the different phenotypes described above per area units. Overall, the quantification of non-COVID-19 lung samples obtained from the tumor-healthy tissue border of lung carcinoma indicated a low number of immune cells (**Figure 4A**, Lung carcinoma, n=5 different individuals with 15-20 serial sections) similar to the healthy lung areas (48, 49). In contrast, a higher infiltration of PMN, lymphocytes, monocytes, and plasma cells in the enhanced coagulation phenotype 1 than lung carcinoma tissues were observed (**Figure 4A**, *p ≤ 0.005 compared to lung carcinoma n=5 different individuals with 15-20 serial sections). These immune cells were mainly distributed in the rim around the hemorrhagic lesions, and only a few cells were observed within the collapsed alveolar space (see **Figure 1** and quantification in **Figure 4A**). In contrast to previous reports, no multinucleated giant cells (MNGC) were detected in all the tissues analyzed (52–54).

A higher immune infiltration was observed for the second phenotype compared to the enhanced coagulation phenotype 1. The overall numbers exceeded at least five times the infiltration observed in the enhanced coagulation phenotype 1, even in the absence of hemorrhagic events, as shown in **Figure 2**. The size of the tissues analyzed was in the range of inches to assure proper unbiased quantification. In the immune infiltration phenotype 2, most of the infiltrated cells corresponded to PMN and monocytes as well as plasma cells supporting a strong cellular and immune response (**Figure 4A**, *p ≤ 0.00021 compared to lung carcinoma tissues, #p ≤ 0.0002 compared to the enhanced coagulation phenotype 1, n=4 different individuals with 15-20 serial sections). Surprisingly, as in enhanced coagulation phenotype 1, a poor T cell influx into the inflamed lungs was observed in the immune infiltration phenotype 2 (**Figure 4A**, lympho). Most of the infiltrated cells were retained or “trapped” inside blood vessels or within the hyperplasic alveolar walls (**Figure 2** and quantification in **Figure 4A**).

In the mixed phenotype 3, despite corresponding to a combination of phenotype 1 and 2, the immune infiltration into the lung was low, probably due to the formation of lung cavities (**Figure 4A**, mixed conditions). The immune infiltration was not well-organized as in the phenotype 1 with smaller hemorrhagic events, lung cavities, and web fibers (**Figure 4A**, Mixed conditions, *p ≤ 0.001 compared to lung carcinoma, n=3 different individuals with 15-20 serial sections and confirmed by confocal microscopy). Overall, each phenotype showed a specific immune infiltration profile.

### Lung Damage Induced by SARS-CoV-2 Is Not Uniform and Denotes the Heterogeneity of the Disease

Despite the variability of the immune and hemorrhagic features described above, we clearly and consistently observed extensive lung damage in all the COVID-19 tissues analyzed. To quantify these structural lung changes, we determined the intravascular and alveolar hemorrhagic events (Coa, for coagulation), the hyperplasia of the alveolar wall (AW), and pneumocytes (HP) as well as the empty area of the lung (EA) and the cell loss of parenchymal cells into the alveolar space (CL) in response to COVID-19 infection using large-scale image analysis. Data is presented in **Figure 4B**. None of these characteristics were observed in the non-COVID-19 lung carcinoma samples or healthy lung (48, 49).

A minimal intravascular RBC accumulation in lung carcinoma tissues was observed (**Figure 4B**, Coa, 10.11 ± 7.02%, the maximal possible value is 100% with only red blood cells in the field). In contrast, the quantification of hemorrhagic events in the enhanced coagulation phenotype indicated a significant increase in hemorrhagic events (69.98 ± 19.84% of the total field) compared to the carcinoma lung tissues, underscoring the large blood accumulation (RBC products) within the lung and the poor surface area available for gas exchange in these individuals (**Figure 4B**, *p ≤ 0.00202 compared to non-COVID-19 lung carcinoma, n=4 different individuals with 15-20 serial sections and confirmed by confocal microscopy). For the second phenotype, immune infiltration, a low blood accumulation was detected compared to the enhanced coagulation condition; however, as indicated above, significant immune infiltration compromised the alveolar space (21 ± 12% of the total field, *p ≤ 0.00101 compared to lung carcinoma, #p ≤ 0.0014 compared to enhanced coagulation n=4 different individuals with 15-20 serial sections). Further, the analysis of mixed conditions indicates that at least half of the lungs had RBC or hemorrhagic events (51.02 ± 11% of the field, *p ≤ 0.00232 compared to lung carcinoma, n=3 different individuals with 15-20 serial sections). Again, these data underscored the different nature of the COVID-19 pathology among different individuals and areas of the lung. The second critical observation was the engrossment of the alveolar walls (AW), the loss of pneumocytes, type I and II, and the overall lung structural lesions (see **Figures 1**–**3** at large magnification).

Normally, the thickness of the alveolar wall is 5.56 ± 3.87 µm. In COVID-19 autopsy lung tissues, alveolar wall thickness was 10.11 ± 7.02 µm (N.S. compared to reported values). Quantification of the alveolar wall thickness was 236 ± 81 µm for the enhanced coagulation phenotype 1, 17.3 ± 6.98 µm for the immune infiltration phenotype 2, and 85 ± 40.2 µm for the mixed phenotype 3 (**Figure 4B**, AW, *p ≤ 0.001 compared to lung carcinoma, #p ≤ 0.0011 compared to enhanced coagulation n=4, different individuals with 15-20 serial sections). However, in addition to the increase in the alveolar wall thickness, more concerning was the loss of key cell types required for gas exchange and the proliferation of accessory cells such as fibroblasts, as we will describe below. In agreement with these concerns, the quantification of the lung’s empty area, a key necessity for the efficient gas exchange, was highly compromised by blood, immune cell infiltration, and fibrosis. Normally, the empty area of the lung corresponds to 83.4 ± 12.34% of the healthy tissue. In agreement, our samples from lung carcinoma showed a 74.83 ± 7.59% of empty lung areas or alveolar space (**Figure 4B**, EA for the empty area). However, only 10.22 ± 2.69% of the total area was empty in the enhanced coagulation phenotype 1, underscoring these patients’ difficulties in achieving efficient gas exchange (**Figure 4**B, EA). In the Immune infiltration phenotype 2, the empty area corresponded to 58 ± 12.65%. The mixed condition phenotype 3, showed 28.98 ± 15.2% (**Figure 4B**, *p ≤ 0.005 compared to lung carcinoma, and #p ≤ 0.05 compared to enhanced coagulation phenotype), suggesting that at in the last two conditions, in theory (see below), some degree of gas exchange can remain.

A critical finding of our histology assessment was the loss of pneumocytes into the alveolar space despite the engrossment of the wall in all cases of COVID-19 analyzed. A similar mechanism of tissue compromise has been observed in Tuberculosis (13, 26, 44, 55–58). Cell loss (CL, for cell loss) in lung carcinoma was minimal to undetectable (0.66 ± 1.184% compared to the cells attached to the alveolar wall, **Figure 4B**, CL, lung carcinoma). In contrast, 80.36 ± 23.35% of the alveolar wall were lost in the enhanced coagulation phenotype (**Figure 4B**, Enhanced coagulation-COVID-19, CL). Also, in the few left un-occluded alveolar walls, a loss of 7.95 ± 4.65% and 5.69 ± 2.36% was observed in the immune infiltration and mixed phenotypes, respectively (**Figure 4B**, AW, *p ≤ 0.0027 compared to Lung carcinoma, #p ≤ 0.0013 compared to enhanced coagulation n=7 different individuals with 15-20 serial sections). These data also underscore the significant damage to the lung, in addition to the hemorrhagic and immune infiltration.

To quantify the hyperplastic nature of pneumocytes in the alveolar wall under COVID-19 conditions (**Figure 4B**, HP), we determined the size of the pneumocytes in uninfected and COVID-19 conditions (phenotypes I and II). In the lung carcinoma samples, the pneumocyte thickness was 4.83 ± 1.09 µm; in contrast, to the enhanced coagulation phenotype, the pneumocyte thickness was 189 ± 78 µm, in the immune infiltration was 25.65 ± 4.98 µm, and the mixed phenotype was 62.2 ± 20.36 µm. These data indicate that COVID-19 increased the size of the pneumocytes and/or enhances the accumulation of collagen or other ECM molecules around them (**Figure 4B**, HP, p ≤ 0.00214 compared to Lung carcinoma, #p ≤ 0.001 compared to enhanced coagulation n=12 different individuals with 15-20 serial sections).

### Fibrosis and Collagen Accumulation, as Well as Compromised Red Blood Cells, Are Uniform Characteristics of the COVID-19 Pathogenesis

We next used trichrome staining to characterize tissue fibrosis and collagen accumulation in the tissues analyzed. As indicated above, each COVID-19 lung phenotype had a unique immune, hemorrhagic, and lung damage characteristic. Some of these characteristics were the formation of intravascular web fibers (fibrin webs, FW), fibrosis inside of the blood vessels (BVC, blood vessel collagen or fibrosis), tissue-associated collagen, or fibrosis (TIC), and the presence of spiked red blood cells (RBCs) (see **Figure 4C**). The only significant differences were observed in blood vessel collagen accumulation in the immune infiltration phenotype compared to the enhanced coagulation and mixed phenotype (**Figure 4C**, *p ≤ 0.00214 compared to lung carcinoma, #p ≤ 0.001 compared to enhanced coagulation n=12 different individuals with 15-20 serial sections).

### COVID-19 Enhances the Infiltration of Macrophages Into the Lung

**Figure 5:**
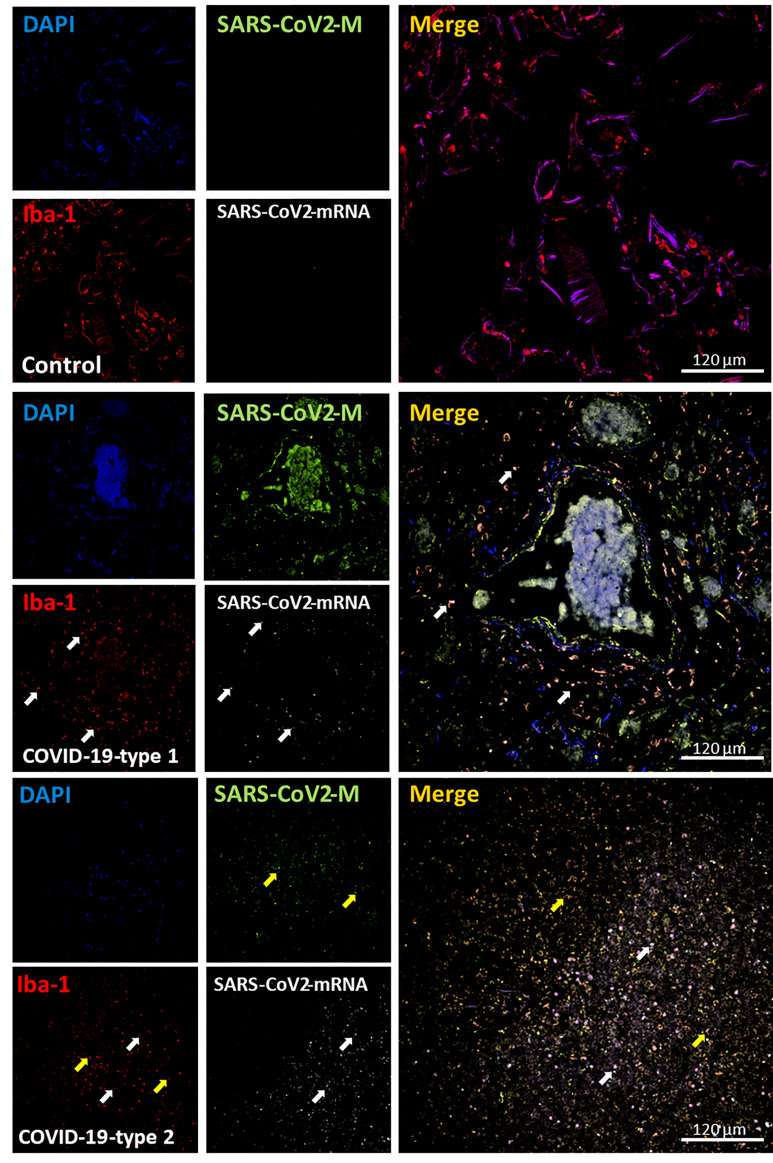
COVID-19 disease induces a significant infiltration into the lung, and a small percentage of macrophages contained viral proteins and mRNA. Staining for nuclei (DAPI, blue staining), SARS-CoV-2 protein M (SARS-CoV-2-M, green staining), Iba-1 (macrophages, red staining), and SARS-CoV-2-mRNA (White staining) was performed. 3D reconstructions of lung samples collected from uninfected (control) and COVID-19 (Type 1 or 2, enhanced coagulation and immune infiltration). In control conditions, macrophages (Iba-1 positive cells) underline the alveolar wall, and no unspecific viral protein or mRNA staining was found as expected (Control). In contrast, the enhanced coagulation phenotype (COVID-19, type I, damage) showed a significant proliferation or migration of macrophages cells (COVID-19-type 1). Also, a small population of Iba-1 positive cells was positive for viral mRNA (see white arrows) and SARS-CoV-2 protein M (yellow arrows). Most viral protein M was concentrated inside blood vessels. In immune infiltrating conditions, also we observed a significant increase in monocyte/macrophage infiltration with a small population of macrophages positive for viral mRNA (white arrow) and protein M without viral mRNA (yellow arrow).

To characterize the immune response against SARS-CoV-2, we performed confocal microscopy and imaging analysis of the subsequent serial sections analyzed by histology to quantify the presence and distribution of viral components and immune cells. Staining for the nucleus (DAPI, blue staining), SARS-CoV-2 protein M (green staining), macrophages (Iba-1, red staining), and SARS-CoV-2-mRNA (S-sense for the newly produced virus) was performed. In uninfected lung carcinoma tissues, macrophages were bigger than in COVID-19 conditions. No SARS-CoV-2 protein or mRNA was detected as expected in the uninfected tissues (**Figure 5**, representative 3D reconstruction/deconvolution of the same tissues analyzed by histology, and **Figure 11A** for the quantification, white arrows indicate Iba-1 positive cells with SARS-CoV-2 mRNA, and yellow arrows indicate Iba-1 positive cells with SARS-CoV-2 protein M but negative for SARS-CoV-2 mRNA). To quantify these images, the staining for Iba-1 was set as 100% in the enhanced coagulation phenotype 1.

In the COVID-19 enhanced coagulation phenotype (**Figure 5** COVID-19-type 1, EC), Iba-1 positive cells accumulate in the parenchymal tissue and surrounded large hemorrhagic events (**Figure 5**, enhanced coagulation phenotype, and 11A). Only a few cells were still positive for SARS-CoV-2 double stranded mRNA, including Iba-1 positive cells, 7.92 ± 3.87%; however, most protein M was located within the blood vessels, suggesting that this viral protein is generated in a different tissue compartment and remains in the circulation for extended periods.

A significant macrophage infiltration was detected in the immune infiltration phenotype 2 (type II, phenotype 2). However, the degree of infiltration was disorganized compared to the enhanced coagulation phenotype (**Figure 5**, COVID-19-type 2, and **Figure 11** for the quantification, *p ≤ 0.0018 compared to lung carcinoma, #p ≤ 0.0011 compared to enhanced coagulation n=7 different individuals with 15-20 serial sections). Tissues from the third phenotype with mixed conditions behave similarly to the immune infiltration phenotype 2 (data not shown, and **Figure 11A**, MX). Overall, macrophage infiltration into the lung was exacerbated in COVID-19 individuals, and macrophage localization and organization depend on the phenotype analyzed. Additionally, we detected a small population of macrophages harboring SARS-CoV-2 mRNA suggestive of local SARS-CoV-2 infection.

### Lymphocytes, CD3 or CD8, Were Poorly Recruited Into the Lung of Fatal COVID-19 Cases

**Figure 6:**
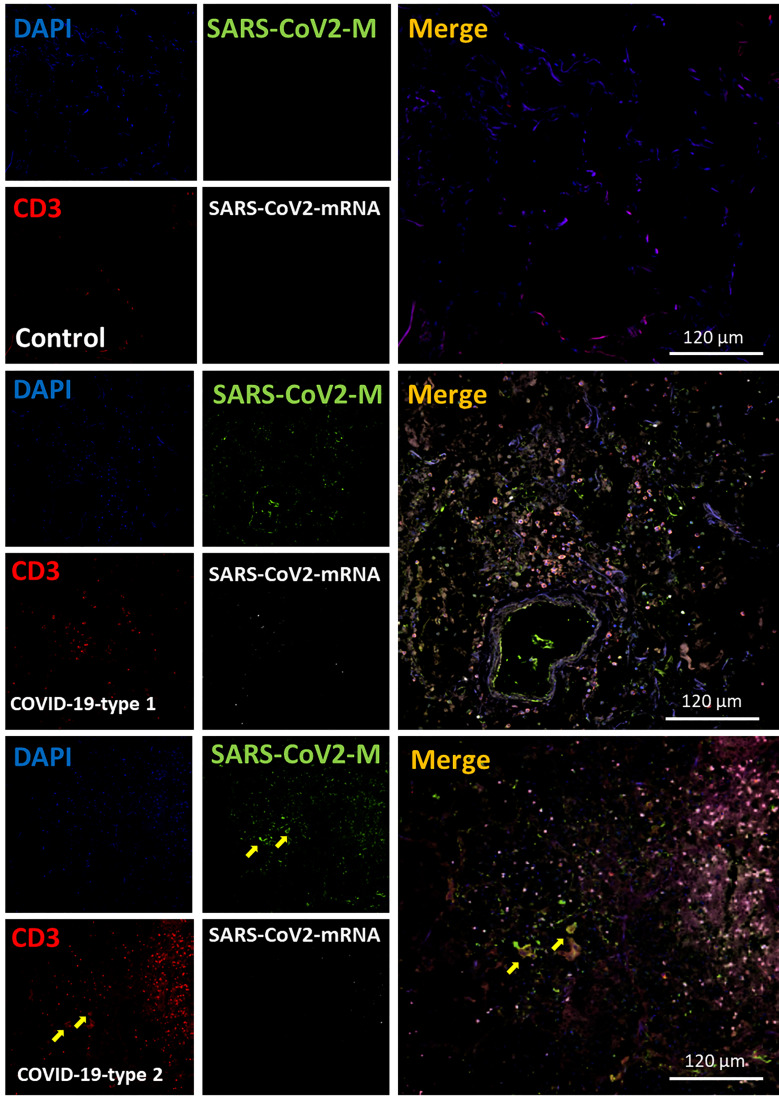
COVID-19 disease induces a small but significant infiltration of CD3 lymphocytes into the lung. Staining for nuclei (DAPI, blue staining), SARS-CoV-2 protein M (SARS-CoV-2-M, green staining), CD3 lymphocytes (CD3, red staining), and SARS-CoV-2-mRNA (White staining) was performed. 3D reconstructions of lung samples collected from uninfected (control) and COVID-19 (Type 1 or 2, enhanced coagulation, and immune infiltration). In control conditions, lymphocytes were mostly randomly attached to the alveolar wall (Control). In contrast, in the enhanced coagulation phenotype (COVID-19, type I, damage), a significant infiltration of lymphocytes was observed (COVID-19-type 1), but most of this infiltration was localized, and most areas were negative for CD3^+^ cells. No CD3^+^ lymphocytes were positive for viral mRNA or the viral protein M. As indicated in the previous figure, most viral protein M staining was concentrated inside blood vessels (see yellow arrows). In the immune infiltrating phenotype, we observed a significant increase in lymphocyte infiltration, but as indicated above, infiltration was region-specific, and most areas were negative for CD3 staining. (n = 13-15 different cases analyzed with a least five sections each).

**Figure 7:**
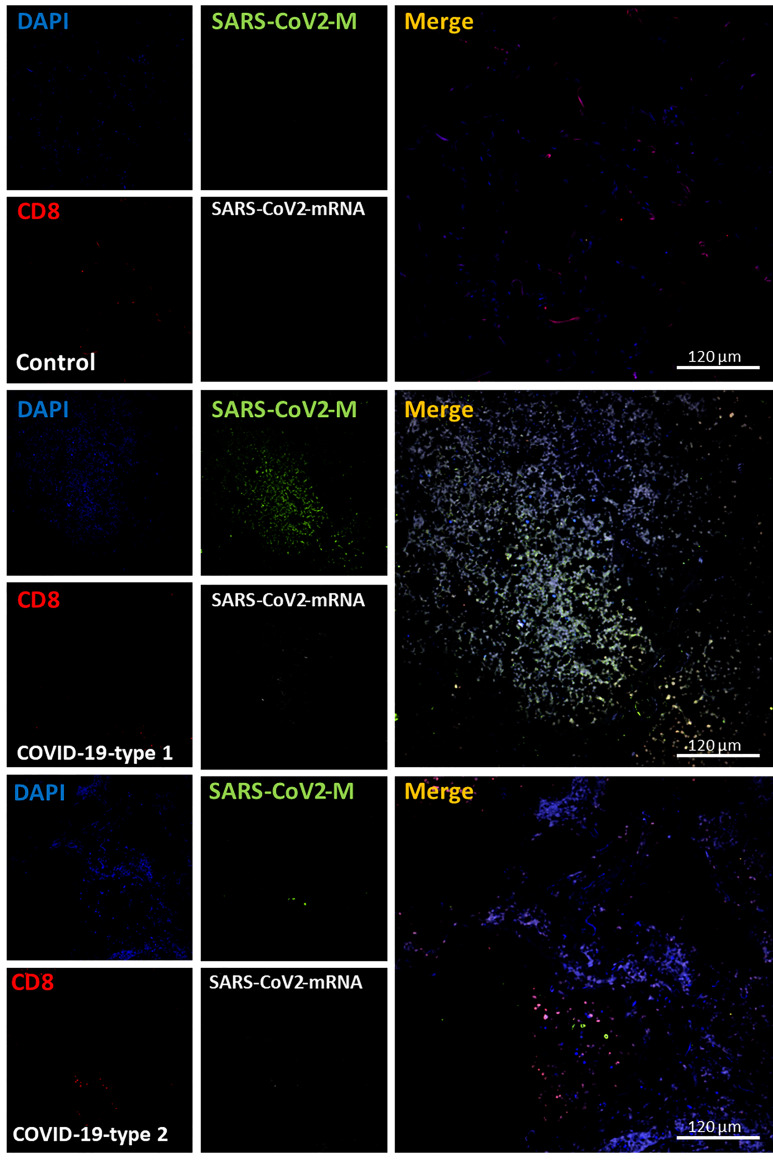
COVID-19 disease induces a small but significant infiltration of CD8^+^ lymphocytes into the lung. Staining for nuclei (DAPI, blue staining), SARS-CoV-2 protein M (SARS-CoV-2-M, green staining), CD8^+^ lymphocytes (CD8, red staining), and SARS-CoV-2-mRNA (White staining) was performed. 3D reconstructions of lung samples collected from uninfected (control) and COVID-19 (Type 1 or 2, enhanced coagulation, and immune infiltration). In control conditions, CD8^+^ lymphocytes were mostly randomly attached to the alveolar wall (Control). In contrast, in the enhanced coagulation phenotype (COVID-19, type I, damage), a significant infiltration of lymphocytes was observed (COVID-19-type 1), but most of this infiltration was localized, and most areas were negative for CD8^+^ cells. No CD8^+^ lymphocytes were positive for viral mRNA or the viral protein M. As indicated in the previous figure, most viral protein M staining was concentrated inside blood vessels. In the immune infiltrating phenotype, we observed a significant increase in lymphocyte infiltration, but as indicated above, infiltration was region-specific, and most areas were negative for CD8 staining. (n = 13-15 different cases analyzed with a least five sections each).

Our histology analysis shows that a small lymphocyte infiltration compared to other inflammatory cells. We performed immune labeling analyzed by confocal microscopy as indicated above, with antibodies to CD3 and CD8 to confirm these data. In lung carcinoma cases, a low number of CD3^+^ and CD8^+^ cells were detected (**Figures 6**, **7** as well as **Figures 11B, C**, CD3 and CD8, respectively). In the case of the CD8 cells, the lung carcinoma cases have increased levels of CD8 infiltrated cells than in COVID-19 cases (*p ≤ 0.0032 compared to Lung carcinoma, #p ≤ 0.00106 compared to enhanced coagulation n=11, different individuals with 15-20 serial sections). Most CD8^+^ cells were also positive for CD3^+^, corresponding to cytotoxic/natural killer T cells (83.06 ± 8.97%) instead of other CD8^+^ cells such as thymocytes and dendritic cells. These data indicate that in at least all the deathly COVID-19 cases analyzed here, a poor CD3^+^ and cytotoxic CD8^+^ response was observed.

### Smooth Muscle Cells Disappeared Upon COVID-19 Infection

**Figure 8:**
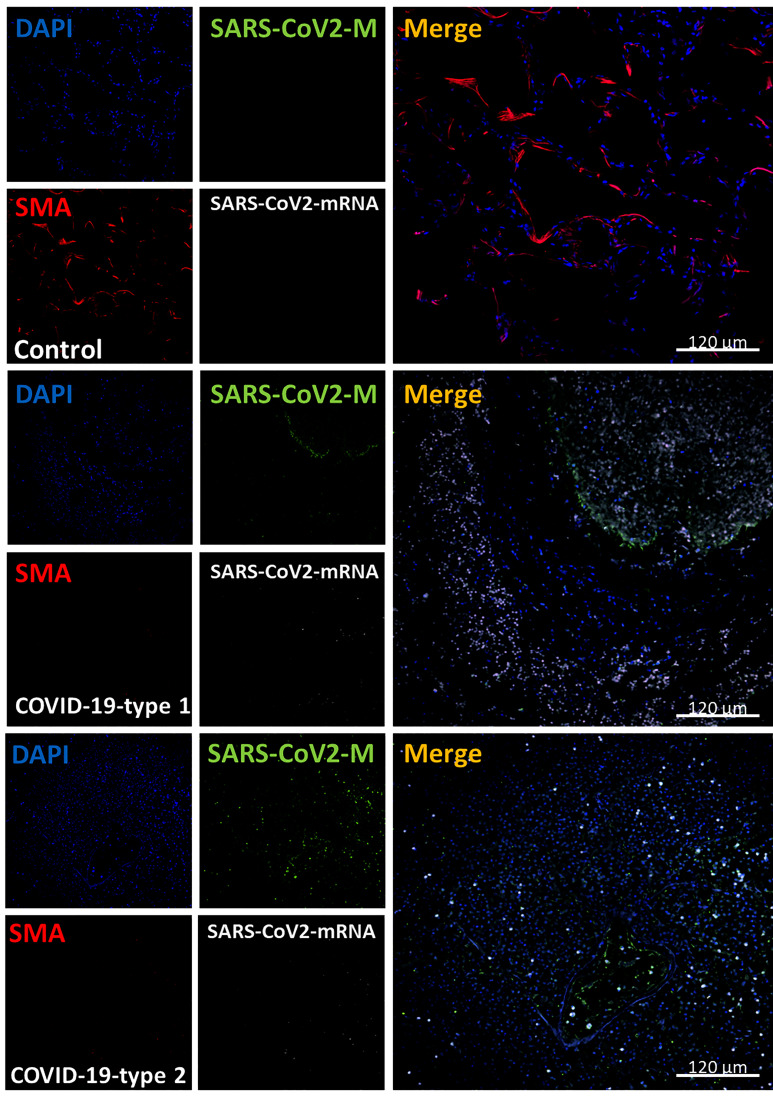
COVID-19 disease induces the total loss of smooth muscle actin cells and lung structure. Staining for nuclei (DAPI, blue staining), SARS-CoV-2 protein M (SARS-CoV-2-M, green staining), smooth muscle actin (SMA, red staining), and SARS-CoV-2-mRNA (White staining) was performed. 3D reconstructions of lung samples collected from uninfected (control) and COVID-19 (Type 1 or 2, enhanced coagulation and immune infiltration phenotype). SMA “decorates” the alveolar wall in control conditions, and no unspecific viral protein or mRNA staining was found as expected (Control). In contrast, in the enhanced coagulation phenotype (COVID-19, type I, damage), SMA’s significant total loss was observed (COVID-19-type 1). Again, most viral protein M was concentrated inside blood vessels. In the immune infiltrating phenotype, we also observed SMC loss in all samples and areas analyzed. (n = 13-15 different cases analyzed with a least five sections each).

Smooth muscle cells (SMC) are a critical contractile component of the airway and an essential contributor to the local production of inflammatory and growth factor products to repair and renew the lung (59–61). To evaluate the SMC distribution and numbers, we performed staining for the nucleus (DAPI, blue staining), SARS-CoV-2 protein M (green staining), smooth muscle actin (SMA, a small muscle marker, red staining), and SARS-CoV-2-mRNA (S-sense for the replicating virus) (**Figure 8**).

In lung carcinoma, smooth muscle cells were observed along blood vessels (**Figure 8**, control, *p ≤ 0.00152 compared to Lung carcinoma, n=13 different individuals with 15-20 serial sections). In contrast, in all the COVID-19 cases analyzed (phenotype 1, 2, and 3), little to no SMA staining was detected in the three types of pathogenic phenotypes, indicating that SMC is lost despite their abundance and importance in lung physiology (**Figures 8**, **11D**). A similar damage pattern was observed in endothelial cells (Von Willebrand positive cells, data not represented). Overall endothelial cells underlying the alveolar wall and the small to medium blood vessels were missing in all COVID-19 cases analyzed (data not shown). These findings add an extra layer to the complexity of the disease because in case these SMCs were lost, the possibilities of recovery and repopulation would be slim to none.

### COVID-19 Increased Fibroblast Proliferation

**Figure 9:**
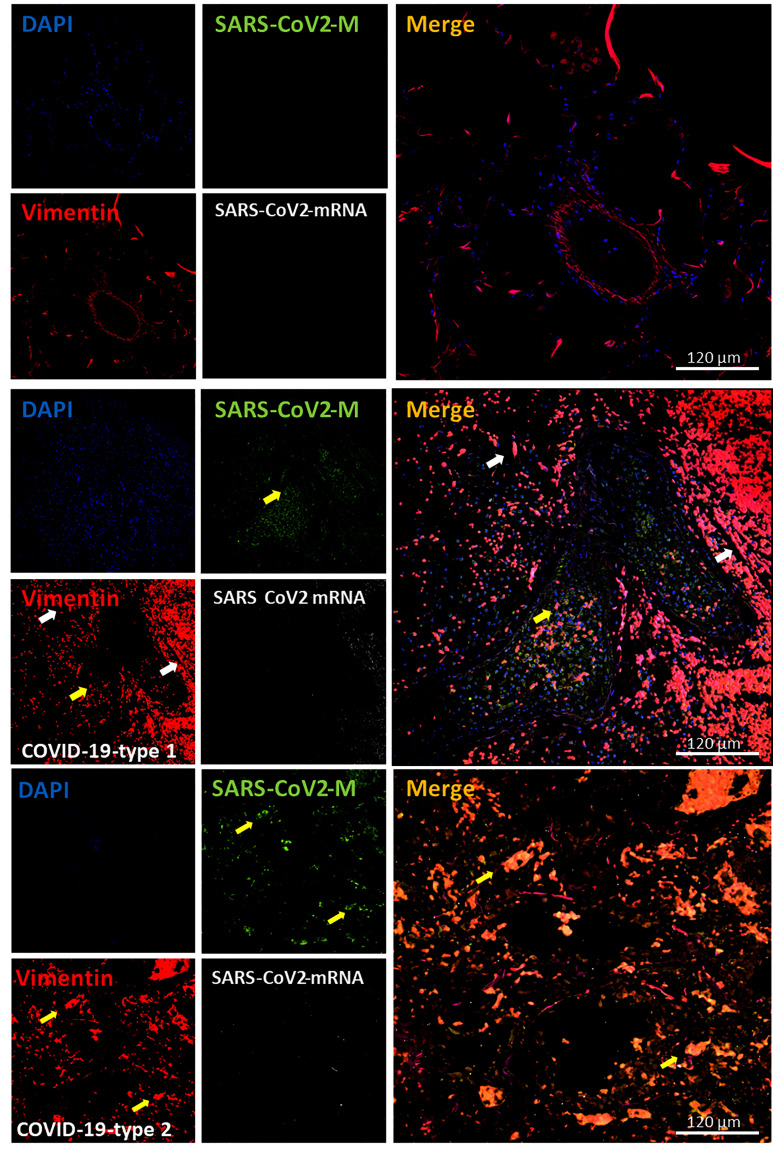
COVID-19 disease induces an exacerbated proliferation of fibroblast. Staining for nuclei (DAPI, blue staining), SARS-CoV-2 protein M (SARS-CoV-2-M, green staining), vimentin (Vimentin, red staining), and SARS-CoV-2-mRNA (White staining) was performed. 3D reconstructions of lung samples collected from uninfected (control) and COVID-19 (Type 1 or 2, enhanced coagulation and immune infiltration phenotype). In control conditions, vimentin-positive cells are located at the alveolar wall base, and around large blood vessels with no unspecific viral protein or mRNA staining were found as expected (Control). In contrast, in the enhanced coagulation phenotype (COVID-19, type I, damage), a large amount of staining widely distributed in the lung was observed (COVID-19-type 1). Again, most viral protein M was concentrated inside blood vessels. Also, a small population of vimentin-positive cells is positive for viral mRNA (see white arrows). Further, a small population of vimentin cells was positive for SARS-CoV-2 protein M without viral mRNA (yellow arrows). We observed a significant proliferation of vimentin-positive cells in all samples and areas analyzed in the immune infiltration phenotype (n = 13-15 different cases analyzed with a least five sections each).

Fibroblasts are the main source of extracellular matrix and fibrosis in pathological conditions. Fibroblasts are not terminally differentiated, and their proliferation is one of their major characteristics upon inflammation. Fibroblasts are positive for vimentin (62–65); thus, we examined its expression, distribution, and positive cell numbers as a readout of fibroblast accumulation within the lung. As indicated in **Figures 9**, **Figure 11E** (quantification), we identified a well-localized vimentin expression on the blood vessels and underling the alveolar wall in uninfected samples (**Figure 9**, Control). However, in all the COVID-19 cases analyzed (n=13), vimentin expression and positive cells significantly increased (**Figures 9**, **11E**, quantification). Most vimentin expression was localized in the parenchyma and surrounding hemorrhagic lesions (**Figure 11E**, *p ≤ 0.00205 compared to lung carcinoma, #p ≤ 0.00185 compared to lung carcinoma to enhanced coagulation n=13 different individuals with 15-20 serial sections). The increase in vimentin expression colocalized perfectly with the increased alveolar thickness observed above, indicating that fibroblasts proliferate and repopulate the alveolar wall despite the pneumocyte loss.

### Epithelial Cells Are Lost in Lung Samples Obtained From COVID-19 Individuals

**Figure 10:**
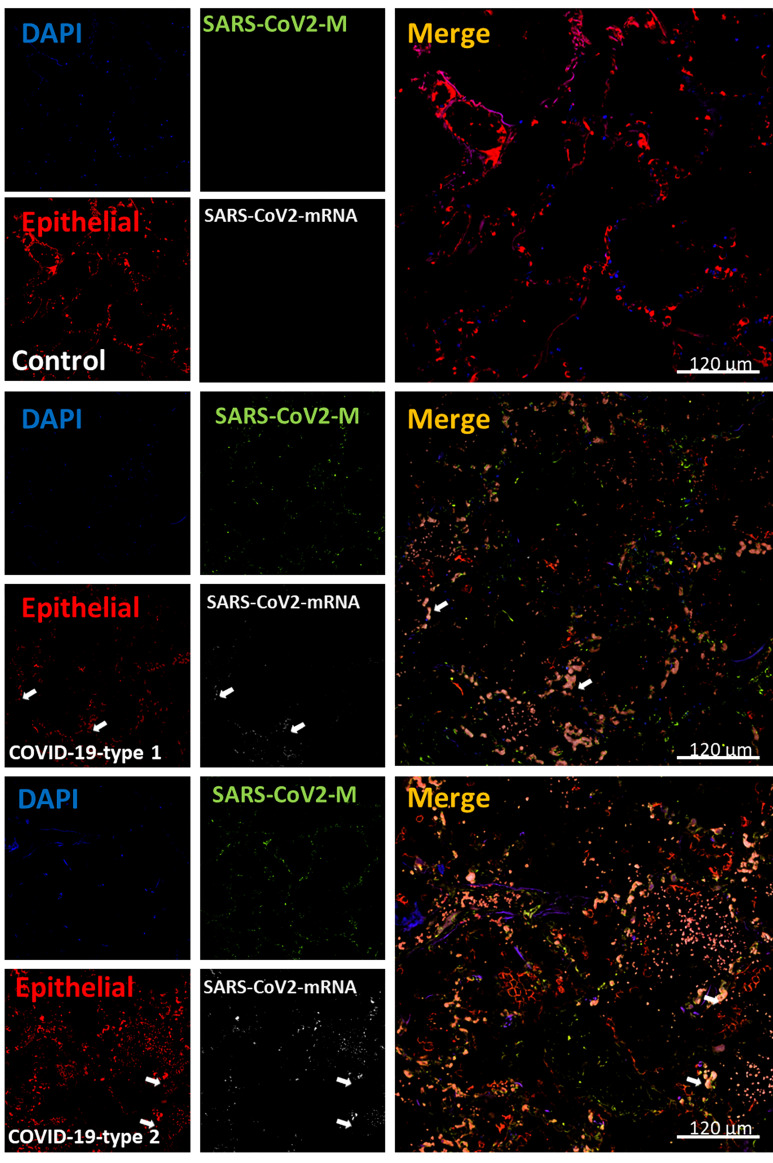
COVID-19 disease induces widespread loss of epithelial cells and compromised polarity, all essential lung function components. Staining for nuclei (DAPI, blue staining), SARS-CoV-2 protein M (SARS-CoV-2-M, green staining), EpCAM (epithelial marker, red staining), and SARS-CoV-2-mRNA (White staining) were performed. 3D reconstructions of lung samples collected from uninfected (control) and COVID-19 (Type 1 or 2, enhanced coagulation and immune infiltration phenotype). In control conditions, EpCAM positive cells underline the alveolar wall as expected (Control). In contrast, in the enhanced coagulation phenotype (COVID-19, type I, damage), a large amount of staining was widely distributed in the lung, but localization was random (COVID-19-type 1). Again, most viral protein M was concentrated inside blood vessels. Also, a population of EpCAM positive cells is positive for viral mRNA (see white arrows). In the immune infiltrating conditions, also we observed significant staining for EpCAM. However, the EpCAM staining was mostly dissociated from the alveolar wall, and groups of epithelial cells can be observed in the alveolar space, even losing their EpCAM polarity, suggesting that cells are probably lost. Thus, the epithelial layer in COVID-19 cases is not functional (n = 13-15 different cases analyzed with a least five sections each).

**Figure 11:**
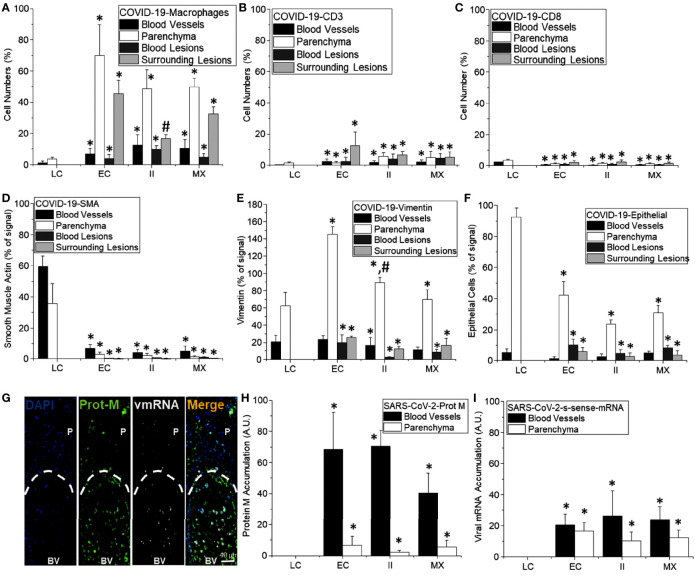
Quantification of cell structure and lung damage in lethal cases of COVID-19. **(A–F)** Correspond to the quantification of different cell types in different compartments of the lung, including blood vessels (black bars), parenchyma (white bars), blood lesions (dark lead-colored bars), and cells surrounding hemorrhagic lesions (lead-colored bars). **(A)** In control conditions (LC for lung carcinoma), minimal numbers of macrophages were detected (mostly resident macrophages). In contrast, in the COVID-19 enhanced coagulation phenotype (EC), a strong infiltration of monocyte/macrophages into the lung parenchyma and around hemorrhagic lesions. Moreover, in the COVID-19 immune infiltration phenotype that lacks hemorrhagic lesions, most macrophages were in the lung parenchyma. (*p ≤ 0.005 compared to lung carcinoma conditions and ^#^p ≤ 0.0001 compared to enhanced coagulation condition, n = 4 different individuals with five sections each). **(B)** Quantification of CD3 cells in different lung compartments as described above. A small and localized CD3 response was found (*p ≤ 0.001 compared to lung carcinoma conditions, n = 13 different individuals with five sections each). **(C)** Quantification of CD8 cells as compared to uninfected samples obtained from individuals with suspected lung carcinoma. Overall, COVID-19 induced a poor CD8 response in the lung in these lethal cases (*p ≤ 0.005 as compared to lung carcinoma conditions, n = 13 different individuals with five sections each). **(D)** Quantification of smooth muscle cells indicates a strong negative effect of COVID-19 in these cells. Overall, SMA or SMC were eliminated by the infection independent of the area analyzed and the damage type induced by COVID-19 infection (*p ≤ 0.001 compared to lung carcinoma conditions, n = 13 different individuals with five sections each). **(E)** Quantification of vimentin-positive cells in COVID-19 lethal cases. Overall, there was a significant and strong increase in Vimentin positive cells, especially in the lung parenchyma (*p ≤ 0.0001 compared to lung carcinoma conditions, n = 13 different individuals with five sections each). **(F)** Quantification of epithelial cells in COVID-19 cases, overall, there is a significant decrease in EpCam staining and numbers of positive cells; however, a critical point is the de-attachment of the remaining epithelial cells from the alveolar wall (*p ≤ 0.0005 compared to lung carcinoma conditions, n = 13 different individuals with 5 section each). **(G)** representative image of the distribution of the viral protein M and the viral mRNA (sense). It is possible to observe the accumulation of these viral components in the circulation and not in the parenchyma (**H, I**, for viral protein M and viral mRNA, respectively. (*p ≤ 1x10^-7^ compared to lung carcinoma conditions, n = 13 different individuals with 5 sections each).

Our histology analysis indicates that large groups of cells are lost into the alveolar space (see **Figures 1**–**3**). To quantify the numbers of epithelial cells, we used an additional antibody to detect epithelial cells, EpCam. In uninfected tissues (control), the distribution of epithelial cells underlines the alveolar wall (**Figure 10**, control). However, in the three COVID-19 phenotypes, the staining for EpCam was lost (**Figures 10**, **11F**), and the few remained epithelial cells were in the alveolar space instead of the alveolar wall (see **Figures 10**, **11F**, *p ≤ 0.00174 compared to lung carcinoma, #p ≤ 0.00106 compared to enhanced coagulation n=13 different individuals with 15-20 serial sections). Epithelial cells in the alveolar space also lost polarized expression and distribution of EpCam. Also, some epithelial cells were still positive for SARS-CoV-2 mRNA as described in other studies (66, 67) (**Figure 10**, see arrows).

### SARS-CoV-2 Protein M Accumulate in Blood Vessels but Poorly in the Lung Parenchyma

We performed confocal and subsequent image analysis to quantify the amount and distribution of protein M. Analysis of blood vessels, and the lung parenchyma indicates that most viral protein M and viral mRNA was associated with the blood vessels or the parenchyma (**Figure 11G**, BV, blood vessels, *versus* P, parenchyma, prot M or mRNA), indicating that most of the viral protein in the three conditions were inside of blood vessels (**Figure 11H**). In contrast, analysis of the viral mRNA in the blood vessels and the lung parenchyma indicates an equal distribution between the two different compartments, independent of the type of inflammation or COVID-19 pathogenesis (**Figure 11I**, *p ≤ 0.00195 compared to lung carcinoma, #p ≤ 0.00143 compared to enhanced coagulation, n=13 different individuals with 15-20 serial sections and confirmed by confocal microscopy). Overall, these data indicated that this viral protein synthesis occurs in another tissue compartment or remains stable in the blood circulation. Still, most of the synthesis in the later stages of COVID-19 did not occur in the lung.

### Overall Assessment of the Clinical Pathology of Deadly COVID-19 Cases

**Figure 12:**
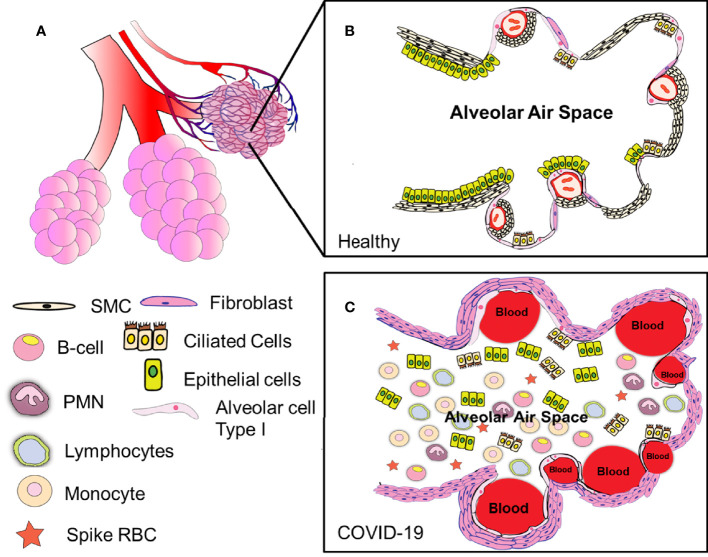
Summary of our findings. Our data indicate that COVID-19 is a highly heterogeneous disease with multiple etiologies **(A)** Correspond to a representation of the alveolar sacs. **(B)** Amplification of the alveolar area to denote the organization of the lung in healthy conditions. **(C)** Correspond to the lung organization in COVID-19 conditions, including enhanced coagulation (intravascular and parenchymal), fibroblast proliferation, loss of epithelial, ciliated, and SMC, as well as endothelial cells from the alveoli. Several questions remain about the immune response, including the lack of immune organization around lesions (as indicated in phenotype 1), the strong B cell response in the lung that could not control the infection, and associated damage. The lack of viral mRNA in the lung and the abundance of protein M inside the blood vessels suggest replication in other tissues. The presence or absence of hemorrhagic events and the formation of fibrin webs inside the blood vessels. All these different phenotypes provide several opportunities to reduce or prevent the devastating consequences of COVID-19.

From our data, COVID-19 appeared as a fast and aggressive multi-visceral disease, with the main lung component having distinct hemorrhagic, immune, and fibrotic phenotypes depending on the patient analyzed. More important was the consistency and widespread parenchymal lung damage in all COVID-19 cases, including loss of endothelial, smooth muscle, and pneumocytes, as well as the exacerbated fibroblast proliferation. Lung parenchymal damage was conserved despite clearly identifying three different kinds of pathology (**Figure 12**). Thus, we propose that cellular products in the lung lavage or saliva could provide essential biomarkers to detect the degree of lung damage and potential treatments.

## Discussion

In 2019, an epidemic outbreak was reported in Wuhan (China) and later was identified as a novel coronavirus named severe acute respiratory syndrome coronavirus 2, SARS-CoV-2, that mainly produces respiratory clinical manifestations (68–71). This highly infectious virus became a worldwide spread pandemic with a 2 to 4% mortality depending on the country and health care conditions (7, 72, 73). Currently, several treatments with limited efficacy and prophylactic vaccines that have reduced efficacy against newly emerging virus variants have been proposed. But a clear mechanistic understanding of this disease still lacks despite the assumption that most COVID-19 induced damage are acute and unique to the SARS-CoV-2. Thus, an urgent understanding of the COVID-19 pathogenesis is required to create new effective treatments based on strong patient-driven data.

Here, we performed an exhaustive characterization of lethal cases of COVID-19 by histology and immune-staining observed by confocal microscopy, accompanied by image analysis. Our data identified three types of lung damage and associated inflammation that underscore the variable nature of COVID-19. The pathology differences among individuals indicate multiple mechanisms of lung damage induced by the virus. Interestingly, our autopsy cases came from individuals with mild high blood pressure, mild BMI, and without diabetes. Our data clearly indicates the lack of a strong T cell response (CD3 and CD8), suggesting an exhausted or compromised immune response. Further, our data underscore the high degree of alveolar wall damage and loss of lung function like no other known disease; the disappearance of smooth muscle cells, endothelial cells, and the pneumocytes, as well as exacerbated fibroblast proliferation, indicate the destructive nature of this virus and the potential long-term consequences in surviving individuals as now observed 20-30% experiencing a long COVID-19. Thus, the pathogenesis of COVID-19 is acute and aggressive and urgently requires new treatments.

However, importantly, as all the cases analyzed corresponded to fatal COVID-19 cases, the damage observed here is likely more limited in patients recovering from COVID-19. Damage resolution would have an additional mechanism of repair and resolution no observed here. Also, the contribution of other tissues or associated general fatal mechanisms cannot be ruled out. Lung compromise is an important part of COVID-19, but other tissues could be important for the fatal consequences and other organ.

A critical point is whether the three different phenotypes of lung damage and immune response to COVID-19 infection are part of the same pathogenic process but represent different disease phases. It is unlikely for the following reasons. First, all the lung damages described involve alveolar damage, enhanced coagulation (intravascular and hemorrhagic events), and fibrosis, corresponded to terminal conditions. Thus, lung damage is variable in different individuals. Second, the enhanced coagulation type 1 of damage shows large hemorrhagic areas that have been “contained” by a strong immune response (immune rim). This phenotype is similar to tuberculosis lesions (39, 74–76). The time required to clear these large hemorrhagic lesions could rank in months to decades. Thus, it is unlikely that this phenotype becomes immune infiltration type 2 or a mixed phenotype 3, or vice-versa in 2-3 weeks, especially with a dysfunctional alveolar wall. Third, the massive damage to the alveolar wall, vasculature, and lung elasticity due to the magnitude of the damage will prevent further immune infiltration and spatial organization formation, as observed in the enhanced coagulation phenotype of COVID-19 disease. Overall, we can conclude that COVID-19 has multiple mechanisms of pathogenesis.

A critical point of all the cases analyzed is the lack of a strong T cell response (CD3 and CD8), suggesting a systemic immune dysfunction. In agreement, a T cell lymphopenia in the blood has been observed in some severe cases of COVID-19 as compared to uninfected individuals (77, 78). Also, the loss of key cell types into the alveolar fluids such as epithelial, smooth muscle, and probably immune cells further compromise the lung and certainly will compromise the exchange of gas and control of pH, both essential components of healthy breathing.

Our studies involving histology, confocal, 3D reconstruction, and deconvolution as well as extensive image analysis indicates multiple differences with early reports analyzing autopsy tissues (16–20), including a spatial resolution to identify cell types and lesions with significant similarities. More important, significant differences in immune infiltration and types of lesions as well as lack of significant viral components (S protein and viral RNA) and multinucleated cells. In addition, we identified critical characteristics of RBC that point to a systemic COVID-19 that might be important for COVID-19 pathogenesis (16–23). Furthermore, the quantification of infiltrated leukocytes indicates a unique inflammatory response that requires further investigation. The exacerbated B cell infiltration without a T cell component and immature leukocyte components suggests an essential role of the bone marrow in COVID-19 pathogenesis. All these points denote the heterogeneous nature of the disease and the multiple types of damage that can be elicited.

A recent publication underscored the strong IFN-α response in circulating proportions of activated T, pro-T, and plasma B cells in the circulation of COVID-19 individuals showing higher cytotoxicity resulting in exhaustion and poor clonal expansion (78). Similarly, we observed a significant RBC sequestration into the lung in the enhanced coagulation phenotype 1 lung type of damage. Despite these differences in the circulation, we did not detect a significant lung infiltration suggesting dysfunction in cellular migration. Thus, tissue-specific recruitment of cell death needs to be examined.

Further, in one published clinical case, inflammatory FCN^+^ macrophages were found to replace FABP4+ macrophages in the bronchoalveolar lavage fluid from severe SARS-CoV-2 infected patients in correlation with a strong CD8^+^ response (79, 80). Total and spike protein-specific T cell responses correlated with spike-specific antibody responses. The group identified 41 peptides containing CD4^+^ and/or CD8^+^ epitopes, including six immunodominant regions. Six optimized CD8^+^ epitopes were defined, with peptide-MHC pentamer-positive cells displaying the central and effector memory phenotype. In mild cases, higher proportions of SARS-CoV-2-specific CD8^+^ T cells were observed. However, a lack of CD8+ response was observed in our cases due to lung structural compromise or lack of proper immune response. Identifying T cell responses in tissue and circulation in severe and milder disease supports the establishment of protective immunity in COVID-19 patients and highlights the potential of including non-spike proteins within future COVID-19 vaccine or treatment design.

An unexpected result was the strong infiltration of B or plasma cells into the lung of COVID-19 infected individuals showing a good local production of antibodies. In COVID-19 patients, unique and specific V(D)J rearrangements in severe patients have been observed, which may be due to an increase of B cell clonality and a skewed use of the IGHV and IGKJ genes (77, 78). However, despite the lung infiltration of plasma cells and probably strong antibody production, none of these patients survived the infection. A potential explanation is a lack of viral components within the lung of these deceased individuals, including mRNA and protein, local TCR activation characterized by a lack of lymphocytes, but the accumulation of viral proteins, especially protein M, into the circulation. Our data demonstrated viral protein M is concentrated inside of the blood vessels as well as viral mRNA; these data suggest that viral replication occurs in different(s) tissue(s) or that viral components are more stable in circulation than in the lung.

We propose that the constant loss of lung cells into the alveolar space can provide multiple biomarkers for lung lavage and saliva detection to design treatments and evaluate lung damage. In our cases, the loss of epithelial cells, smooth muscle cells, and the exacerbated proliferation of fibroblasts represent the irreversible nature and stage of the disease. The examination of recovered cases will be essential to understand the degree and long-term damage. The analysis of mild COVID-19 cases could provide essential information for future healthcare. We believe that endothelial/alveolar compromise is a key element that prevents immune cells from migrating into the lung’s compromised areas to achieve resolution. Thus, in surviving individuals, probably long-term care and recovery may be considered in the near future.

In conclusion, our data offers a novel and more complex view of the COVID-19 pathogenesis and contributes to new insights into treatments and the identification of biomarkers that prevent or predict the onset of this devastating disease.

## Data Availability Statement

The raw data supporting the conclusions of this article will be made available by the authors, without undue reservation.

## Author Contributions 

All authors contributed to performing the experiments, analyzing the data, and writing the manuscript. All authors contributed to the article and approved the submitted version.

## Funding

This work was supported by a Flash COVID fund from both ANR and Fondation pour la Recherche Medicale (ANR-20-COVI-000 and AO2020- MUCOLUNG-ANR Flash COVID-19 - FRM) to MB. This work was funded by The National Institute of Mental Health grant, MH096625 and MH128082, the National Institute of Neurological Disorders and Stroke, NS105584, and UTMB internal and State of Texas funding (to EE).

## Conflict of Interest

The authors declare that the research was conducted in the absence of any commercial or financial relationships that could be construed as a potential conflict of interest.

## Publisher’s Note

All claims expressed in this article are solely those of the authors and do not necessarily represent those of their affiliated organizations, or those of the publisher, the editors and the reviewers. Any product that may be evaluated in this article, or claim that may be made by its manufacturer, is not guaranteed or endorsed by the publisher.

## Abbreviation

FDA, Food and Drug Administration; PBMCs, peripheral blood mononuclear cells; PBS, Phosphate-buffered saline; SARS-CoV-2, severe acute respiratory syndrome coronavirus-2; COVID-19, Coronavirus disease 2019; CD, cluster of differentiation; TB, tuberculosis; SMA, Smooth Muscle Actin; MPO, myeloperoxidase; EpCam, epithelial cam; H&E, hematoxylin and eosin; PMN, polymorphonuclears; FW, fibrin webs; RBC, red blood cells; LC, lung carcinoma; lympho, lymphocytes; monocytes, mono; plasma cells or B cells (Plasma); MNGC, multinucleated giant cells; Coa, coagulation; alveolar wall, AW; hyperplasia pneumocytes, HP; empty lung area, EA; cell loss, CL; DAPI, 4′,6-diamidino-2-phenylindole; BV, blood vessels; P, parenchyma; TCR, T cell receptor; BMI, body mass index; BVC, blood vessel collagen; TIC, Tissue associated collagen.
