# Supplementary material for: COVID-19 Lung Pathogenesis in SARS-CoV-2 Autopsy Cases
Source: Front Immunol. 2021 Oct 4;12:735922. doi: 10.3389/fimmu.2021.735922 (PMC8521087; doi:10.3389/fimmu.2021.735922)
Supplement: Supplementary file 1 [file DataSheet_1.pdf]

COVID-19 lung pathogenesis in SARS-CoV2 autopsy cases

Valdebenito Silvana[1], Bessis Simon [2], Djillali Annane[3,4,5], Geoffroy Lorin de Grandmaison[6], Cramer Bordé-Elisabeth [7], Prideaux Brendan [1], Eugenin A. Eliseo[1,*], and Bomsel Morgane[8, *]

Affiliations: [1]Department of Neuroscience, Cell Biology, and Anatomy, University of Texas Medical Branch (UTMB), Galveston, Texas, USA. [2]Service des Maladies Infectieuses, Centre Hospitalier Universitaire Raymond Poincaré, AP-HP, Garches, France. [3]Intensive Care Unit, Raymond Poincaré Hospital (AP-HP), Paris, France. [4]Simone Veil School of Medicine, Université of Versailles, Versailles, France. [5]University Paris Saclay, Garches, France. [6]Department of Forensic Medicine and Pathology, Versailles Saint-Quentin Université, AP-HP, Raymond Poincaré Hospital, Garches, France. [7]University of Versailles Saint Quentin en Yveline, France. [8]Laboratory of Mucosal Entry of HIV-1 and Mucosal Immunity, Department of Infection, Immunity, and Inflammation, Cochin Institute, CNRS (UMR 8104), Paris, France.

* Corresponding author and lead contact: Dr. Morgane Bomsel. Mucosal Entry of HIV and Mucosal Immunity, Institute COCHIN, University of Paris, Paris, France., email: morgane.bomsel@inserm.fr or Dr. Eliseo Eugenin Ph.D., Department of Neuroscience, Cell Biology, and Anatomy, University of Texas Medical Branch (UTMB), Research Building 17, 105 11th Street, Galveston, Texas, 77555, USA. Phone, 409-772-7705, Email: eleugeni@UTMB.edu

Running Title: COVID-19 and lung pathogenesis

Keywords: COVID-19; cytokine storm; lung; virus; SARS

Supplemental Figures.

Supplemental Figure 1: Representative microphotograph of eight serial sections of lung obtained from deadly cases of suspicious lung carcinoma cases. Compare to Fig. 1. (A) Representative H&E stained images of lung obtained from lung carcinoma samples. Low magnification allows us to appreciate the normal alveolar wall and minimal intravascular coagulation. Most cells are denoted (monocytes and plasma cells, blue and yellow arrows) with arrows correspond to the lung's resident cells. Bar: 2.5 mm. (B-J) Correspond to a higher magnification of the healthy lung parenchyma: bar 100 μm and 25 μm. Compare to Fig. 1. No damage was detected in these samples. n=4 different individuals with 15-20 serial sections.

Supplemental Figure 2: Representative microphotograph of eight serial sections of lung obtained from lung carcinoma and stained for trichome staining. Compare supplemental figure 1. (A) Representative trichrome staining images of supplemental Fig. 1. Low magnification allows us to appreciate the high amount of staining versus the damage observed in Fig. 1. Bar: 2.5 mm. (B-J) Correspond to a higher magnification of the healthy lung parenchyma: bar 100 μm and 25 μm. Compare to supplemental Fig. 1. No damage was detected in these samples. n=4 different individuals with 15-20 serial sections.

Supplemental Figure 3: Representative microphotograph of eight serial sections of lung obtained from a deadly case of COVID-19 and stained for trichome staining,

enhanced coagulation phenotype. Compare supplemental figures 1 and 2, COVID-19 versus Normal lung. (A) Low magnification allows us to appreciate the high amount of staining versus the damage observed in Fig. 1. Bar: 5 mm. (B-J) Correspond to a higher magnification of the healthy lung parenchyma: bar 100 μm and 25 μm. Compare to Fig. 1. No damage was detected in these samples. n=4 different individuals with 15-20 serial sections.

Supplemental Figure 4: Representative microphotograph of eight serial sections of lung obtained from a deadly case of COVID-19 and stained for trichome staining, immune infiltration. Compare to Figure 2. (A) Representative trichrome staining images of Fig. 2. Low magnification allows us to appreciate the high amount of staining versus the damage observed in Fig. 2. Bar: 5 mm. (B-J) Correspond to a higher magnification of the COVID-19 lung with immune infiltration phenotype: bar 100 μm and 25 μm. Compare to Fig. 2. n=4 different individuals with 15-20 serial sections.

Supplemental Figure 5: Representative microphotograph of eight serial sections of lung obtained from a deadly case of COVID-19 and stained for trichome staining, Mixed conditions. Compare to Figure 3. (A) Representative trichrome staining images of Fig. 3. Low magnification allows us to appreciate the high amount of staining versus the damage observed in Fig. 3. Bar: 2.5 mm. (B-J) Correspond to a higher magnification of the COVID-19 lung with a mixed phenotype: bar 100 μm and 25 μm. Compare to Fig. 3. The damage was associated with immune

infiltration and hemorrhagic conditions. n=4 different individuals with 15-20 serial sections.

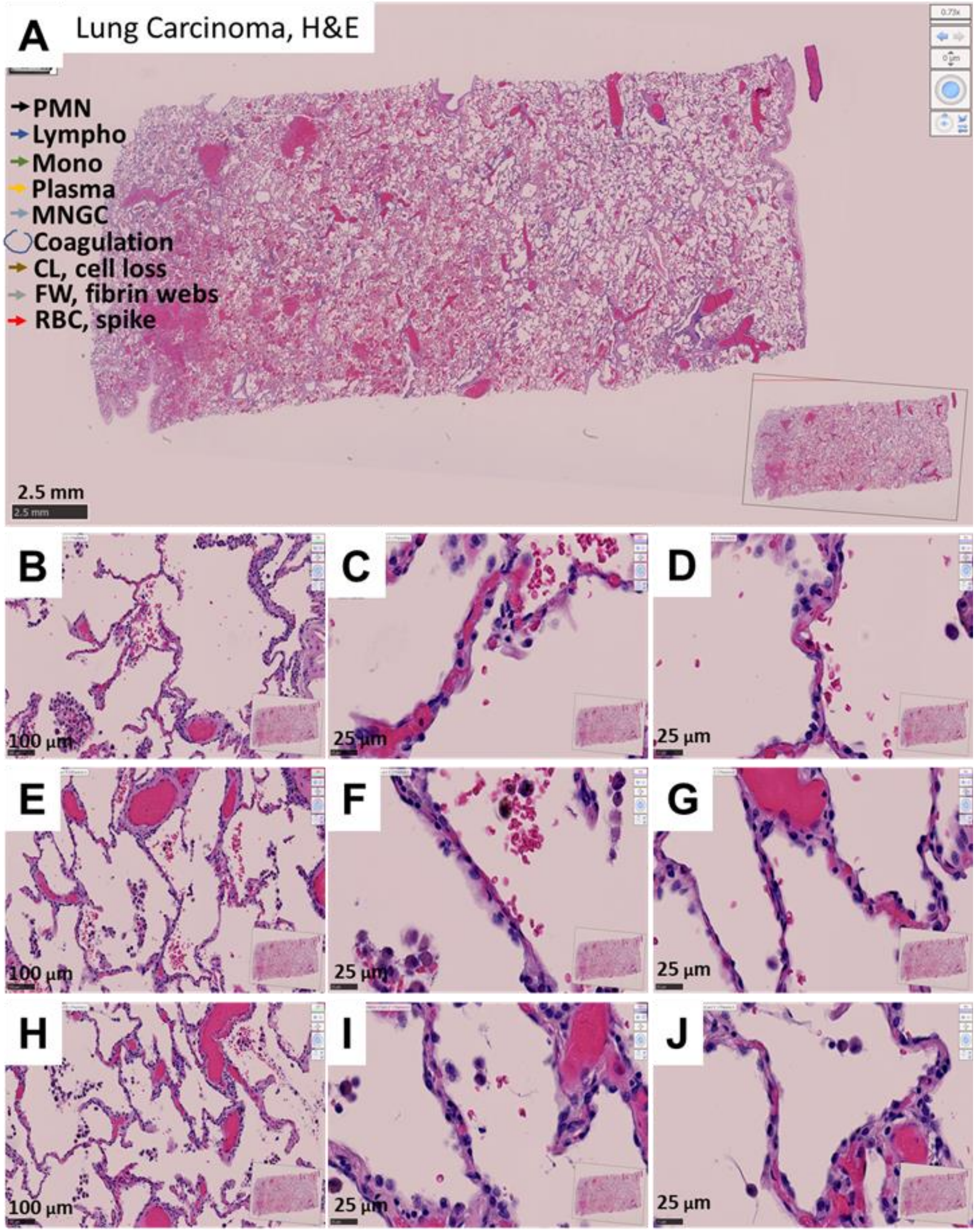

Valdebenito et al., Supplemental Figure 1

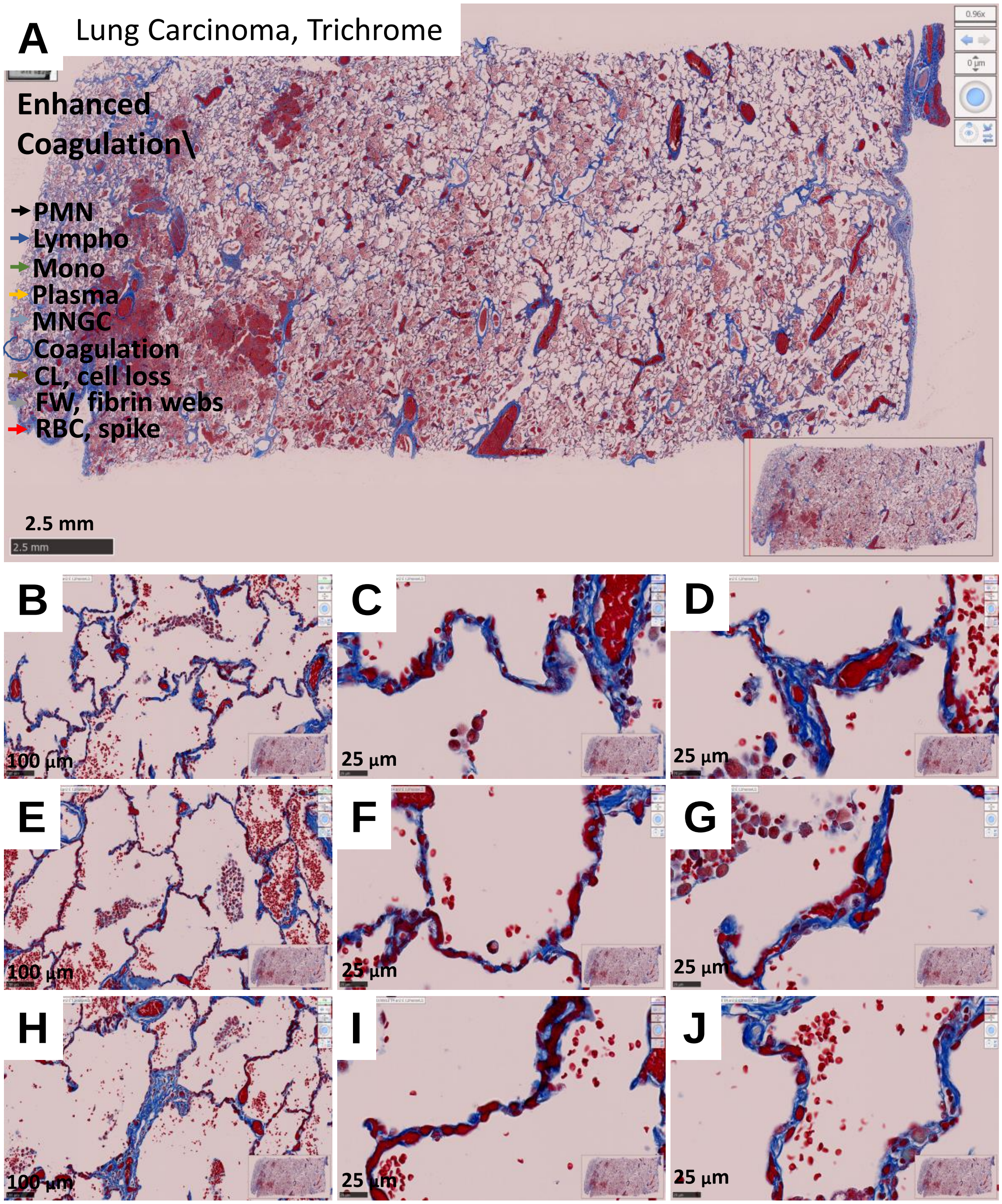

Valdebenito et al., Supplemental Figure 2

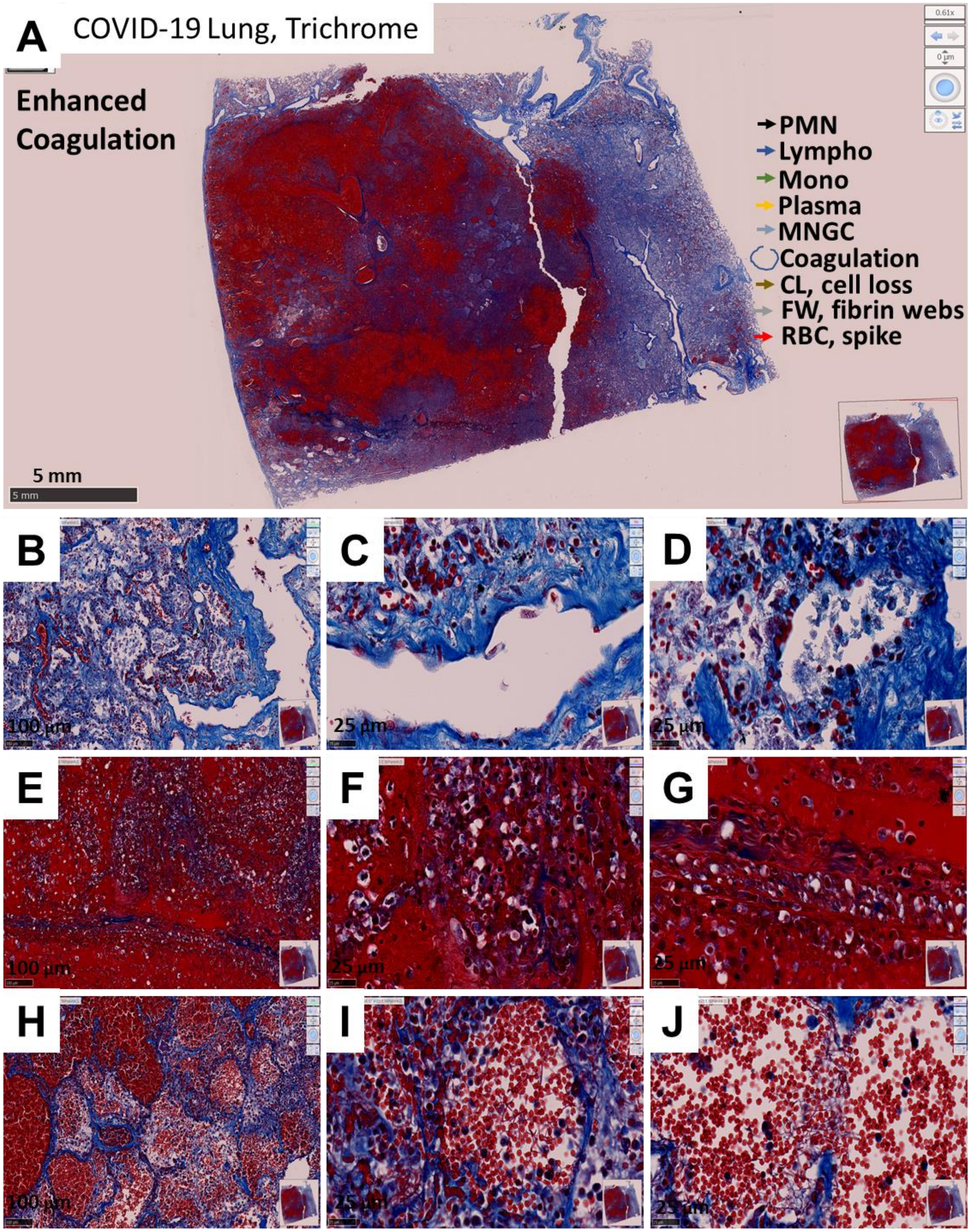

Valdebenito et al., Supplemental Figure 3

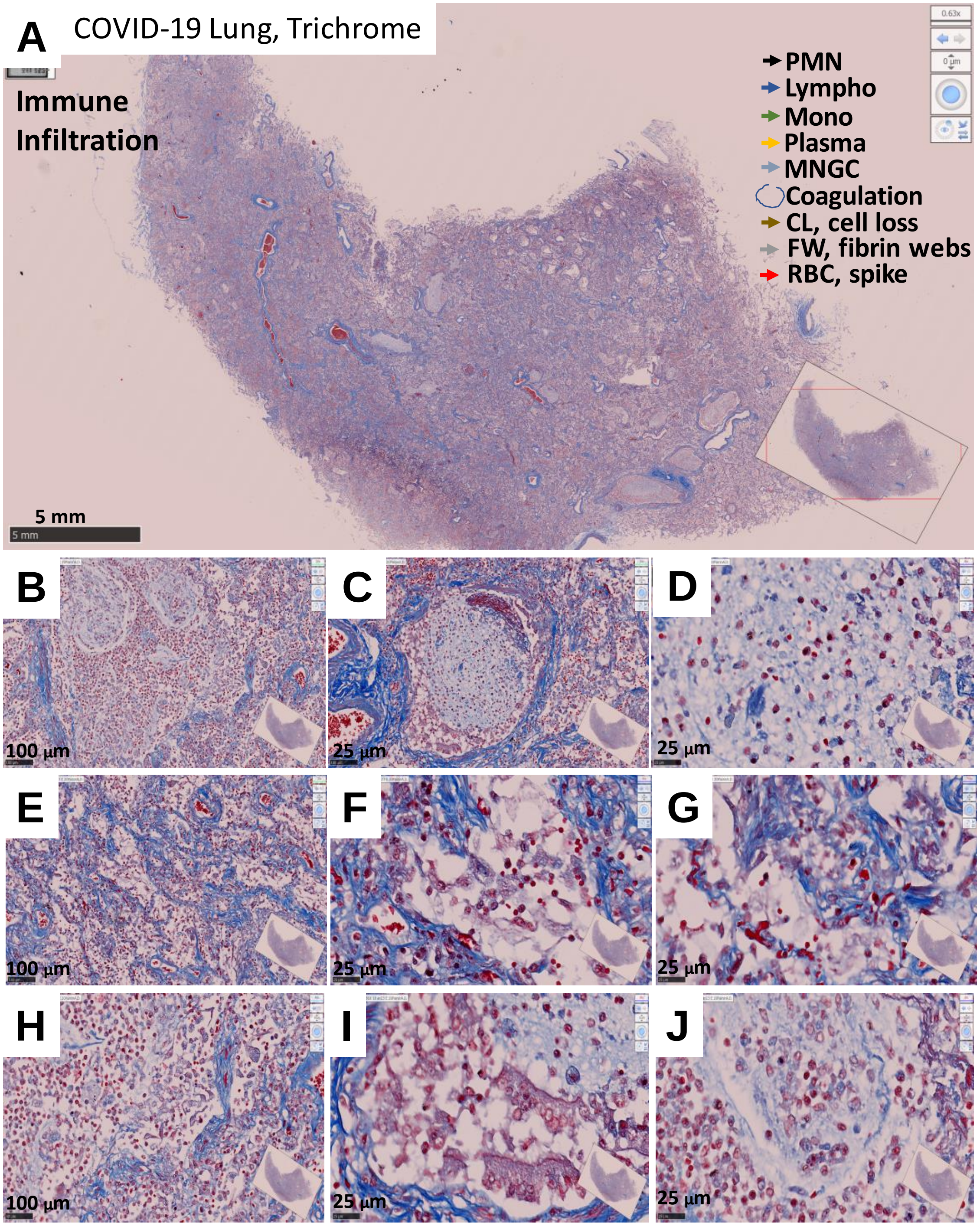

Valdebenito et al., Supplemental Figure 4

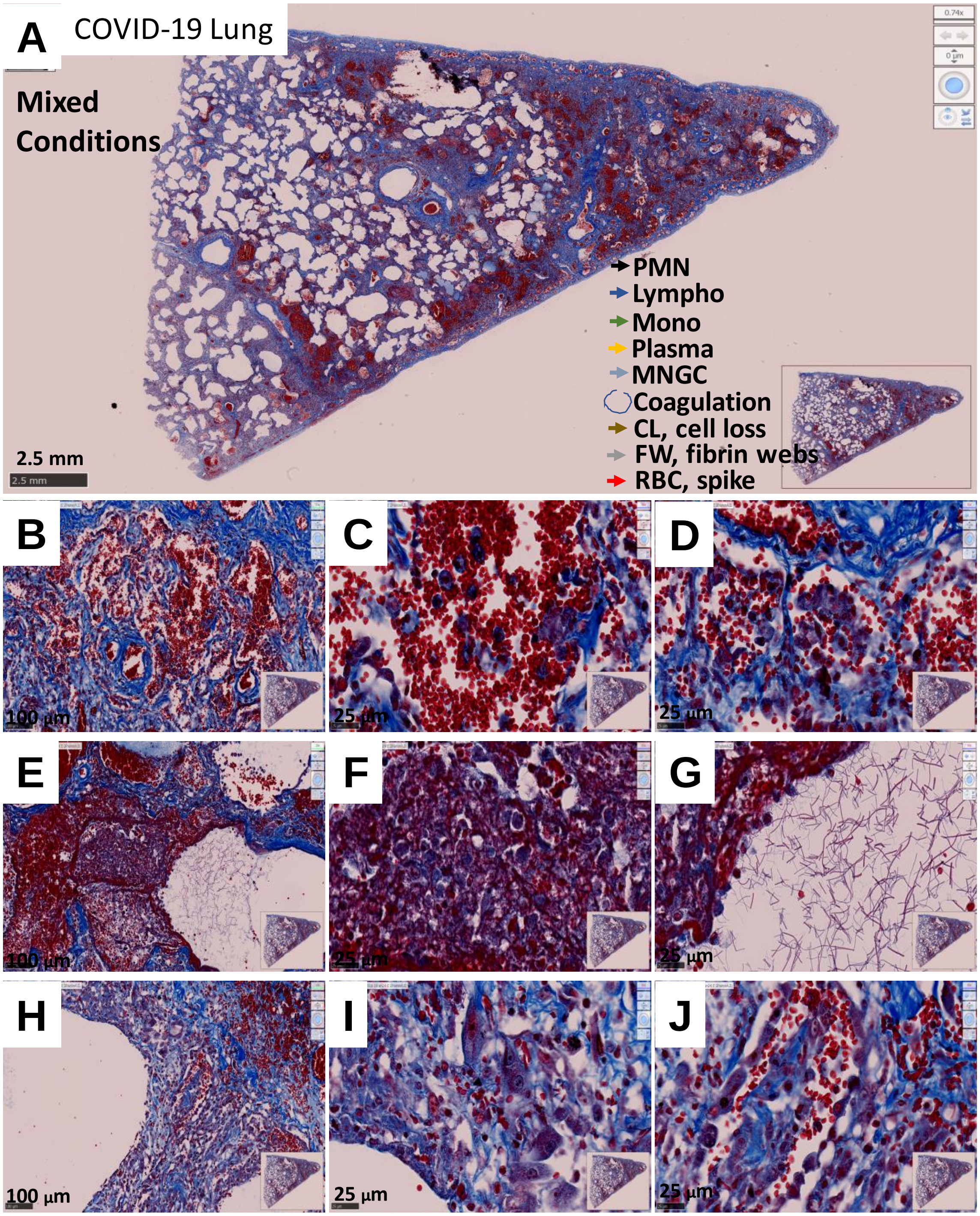

Valdebenito et al., Supplemental Figure 5
